# Synthesis, characterization, and biological activities of zinc(II), copper(II) and nickel(II) complexes of an aminoquinoline derivative

**DOI:** 10.3389/fchem.2022.1053532

**Published:** 2022-11-03

**Authors:** Tadewos Damena, Mamaru Bitew Alem, Digafie Zeleke, Tegene Desalegn, Rajalakshmanan Eswaramoorthy, Taye B. Demissie

**Affiliations:** ^1^ Department of Applied Chemistry, Adama Science and Technology University, Adama, Ethiopia; ^2^ Department of Chemistry, Wachemo University, Hossana, Ethiopia; ^3^ Department of Chemistry, Salale University, Fitche, Ethiopia; ^4^ Department of Biomaterials, Saveetha University, Chennai, India; ^5^ Department of Chemistry, University of Botswana, Gaborone, Botswana

**Keywords:** aminoquinoline, novel metal complexes, DFT analysis, molecular docking, antibacterial, antioxidant

## Abstract

Interest is increasingly focused on the use of transition metal complexes as biochemical, medical, analytical, pharmaceutical, agronomic, anticancer, and antibacterial agents. In this study, three complexes of [Zn(H_2_L)Cl] (**1**), [Cu(H_2_L)(H_2_O)(NO_3_)] (**2**) and [Ni(H_2_L)(NO_3_)].2H_2_O (**3**) were synthesized from a 2-chloroquinoline-3-carbaldehyde derived ligand [H_3_L = ((*E*)-2-(((2-((2-hydroxyethyl)amino)quinolin-3-yl)methylene)amino)ethanol. The compounds were characterized using physicochemical and spectroscopic methods. The results demonstrate that the free ligand behaves as a tridentate ligand with one oxygen and two nitrogen (ONN) donor atoms in 1:1 metal:ligand ratio. The formation constants of the complexes were found to be (*K*_Zn(II)_ = 2.3 × 10^6^, *K*_Cu(II)_ = 2.9 × 10^6^, *and K*_Ni(II)_ = 3.8 × 10^5^). The thermodynamic parameters indicated that the reactions were spontaneous with exothermic nature of metal-ligand interaction energies. Based on the analyses of the experimental (EDX, FTIR, PXRD, MS and TGA) and DFT results, a distorted tetrahedral, a distorted square pyramidal and square planar geometry for Zn(II), Cu(II) and Ni(II) complexes, respectively, were proposed. The B3LYP calculated IR frequencies and TD-B3LYP calculated absorption spectra were found to be in good agreement with the corresponding experimental results. The powder XRD data confirmed that the Zn(II), Cu(II) and Ni(II) complexes have polycrystalline nature with average crystallite sizes of 27.86, 33.54, 37.40 Å, respectively. *In vitro* antibacterial activity analyses of the complexes were studied with disk diffusion method, in which the complexes showed better activity than the precursor ligand. Particularly the Cu(II) complex showed higher percent activity index (62, 90%), than both Zn(II) (54, 82%) and Ni(II) (41, 68%) complexes against both *E. coli* and *P. aeruginosa*, respectively. Using the DPPH assay, the complexes were further assessed for their antioxidant capacities. All metal complexes showed improved antioxidant activity than the free ligand. Zn(II) and Cu(II) complexes, which had IC_50_ values of 10.46 and 8.62 μg/ml, respectively, showed the best antioxidant activity. The calculated results of Lipinski’s rule of five also showed that the target complexes have drug-like molecular nature and similarly, the results of binding mode of action of these compounds against *E. coli* DNA gyrase B and *P. aeruginosa* LasR.DNA were found to be in good agreement with the *in vitro* biological activities.

## 1 Introduction

The application of transition metal complexes as biochemical, medicinal, analytical, pharmaceutical, agricultural, antitumor and antimicrobial agents has become center of interest for researchers (Fetoh et al., 2018; Hamdani and Amane, 2019; Sumalatha et al., 2021). Recently, huge focus is being geared towards the chemistry of coordination compounds due to the inherent potential of metals and their organic based complexes for treatment of various health problems and disorders (Fetoh et al., 2018; Hamdani and Amane, 2019). In this regard, the syntheses of biologically active complexes have paramount significance. Research results over the past decades have witnessed that metal complexes were found to exhibit potential antimicrobial and antioxidant (El-Gammal et al., 2021; Kargar et al., 2021; Kaya et al., 2021; Damena et al., 2022a), antiviral (Atasever Arslan et al., 2021), anticancer (Malik et al., 2021), antidiabetic (Koleša-Dobravc et al., 2018) and cytotoxicity (Alem et al., 2022) activities. In this aspect, the ongoing search for natural products with potential biologically active ligands have confirmed that quinoline and its derivative ligands lie among the important classes of biologically active ligands. Such ligands have become interesting due to their extensive pharmacological properties and applications, such as anticancer (Fouda, 2017), antifungal and antiprotozoal (Ramírez–Prada et al., 2017), anti-inflammatory (Pinz et al., 2017), antidiabetic (Murugavel et al., 2017), antimicrobial (Nagesh et al., 2015; Digafie et al., 2021; Damena et al., 2022a), and antioxidant activities (Digafie et al., 2021; Halevas et al., 2021; Damena et al., 2022a).

Previous studies show that zinc complexes exhibit antidiabetic (Koleša-Dobravc et al., 2018), antioxidant (Halevas et al., 2021; Damena et al., 2022a), and antimicrobial activities (Nagesh et al., 2015; Damena et al., 2022a), whereas copper (II) and nickel (II) complexes have antimicrobial, antioxidant, DNA binding, and antiviral activities (Zou et al., 2017; Atasever Arslan et al., 2021; El-Gammal et al., 2021; Kargar et al., 2021). However, the structural and biological properties of metal complexes with imine containing N-heterocyclic ligand, [H_3_L= ((*E*)-2-(((2-((2-hydroxyethyl)amino)quinolin-3-yl)methylene)amino)ethanol, have not been reported. Hence, we hereby report the synthesis of these metal complexes from the corresponding metal salts (zinc chloride, copper nitrate trihydrate, and nickel nitrate hexahydrate with their biological (antibacterial and antioxidant) properties using disc diffusion and DPPH assay methods, respectively. Furthermore, computational studies had been performed to better understand the properties and activities of the complexes and to correlate with the experimental results.

## 2 Experimental methods

### 2.1 Materials

Acetic anhydride 99.8%, acetic acid glacial 99.5%, aniline 99%, N, N- dimethyl formamide 99%, phosphorus oxychloride 98%, methanol 99.5%, n-Hexane 99%, dichloromethane 98%, Ethyl acetate 99.5%, chloroform 99%, Triethylamine 99%, copper nitrate trihydrate 98%, nickel nitrate hexahydrate 98%, zinc chloride 98%, silver nitrate 99.9%, L-Ascorbic acid 99%, Dimethyl sulphoxide 99% and 2, 2-diphenyl-1-picrylhydrazyl (DPPH) were used. All the chemicals and reagents, analytical grade, were purchased from Loba chemie PVT. LtD (Mumbai, India).

### 2.2 Characterization techniques

The NMR spectra of the ligand were obtained using NMR Bruker Avance 400 spectrometer operating at 400 MHz using DMSO-d6 and CDCl_3_. Chemical shifts (δ) are reported in ppm and the coupling constants (*J*) are reported in Hz. Fluorescence and UV-visible spectral data were measured using Agilent MY-18490002/PC spectrofluorophotometer and SM-1600 Spectrophotometer, respectively. The absorption spectra of the synthesized compounds were measured using 1.0 × 10^–5^ M methanolic diluted solution. Elemental composition and morphology were carried out using scanning electron microscopy with energy dispersive X-ray (SEM-EDX, CARL ZE 155, OXFORD instrument’s EDX, USA Hitachi SU 70 Oxford Instruments 50 mm^2^ X-Max silicon drift EDS detector, with resolution of 127 eV FWHM and detection limit of about 1 atomic % from depth of 0.3–3 μm). Mass spectra were recorded with SHIMADZU, LC-MS 8030 (model LCMS-8030, mass range *m/z* 10 to 2000, sensitivity resolution R < 0.7 FWHM). X-ray diffractometer (SHIMADZU model: XRD-7000 X-RAY DIFFRACTOMETER) was used to measure the X-ray diffraction. The patterns of the synthesized metal complexes were performed using powder sample with measurement conditions of X-ray tube target: Cu (*λ* = 1.5406 Å), voltage: 40.0 kV, current: 30.0 mA, divergence slit: 1.0^o^, scatter slit: 1.0^o^, receiving slit: 0.3 mm, scanning drive axis: 2θ, scan range: 5.0–80.0^o^, scan mode: continuous scan, Scan speed: 3.0 ^o^/min, sampling pitch: 0.02^o^.

The Chekcell Graphical Powder Indexing (CCP14) program was used to calculate the miller indices, whereas the lattice parameters were determined using the CRYSFIR computer program. FTIR measurements were performed using Perkin-Elmer BX spectrometer (from 4000–400 cm^−1^ and KBr pellets). Thermogravimetric analyses (TGA) data were recorded using DTG-60H SHIMADZU thermal analyzer under nitrogen-atmosphere (20 ml/min) and heating rate of 10^°^C/min between 25–800°C. Molar conductance of the complexes were recorded at room temperature in 1 × 10^–3^ M methanolic solution of the samples using conductometre (AD8000: resolution 0.1 mV (±699.9 mV)/1 mV (±2000 mV), 0.01, 0.1, 1 μS/cm; ppm, 0.01, 0.1 mS/cm; ppt 0.1°C, accuracy at 25°C ± 0.2 mV up to ±699.9 mV, ± 1 mV up to ±2000 mV), ±0.5°C). Melting point analysis was performed using digital auto melting point apparatus (Hanchen, model 934).

### 2.3 General procedure for the synthesis of the ligand (H_3_L)

The ligand was prepared based on our previously reported procedure (Digafie et al., 2021) with minor modifications, in which 2-chloroquinoline-3-carbaldehyde (2.5 g, 0.013 mol) was added to 15 ml ethanol amine and refluxed at temperature ranges of 90–95°C for 2 h. The progress and completion of the reaction was monitored using TLC. After completion, the resulting mixture was cooled and then put into crushed ice. The precipitate was collected through suction filtration and washed with ice cold water to remove the excess amount of ethanolamine which served both as solvent and reagent as well as unreacted material, and finally dried at room temperature (Digafie et al., 2021). The ligand has a molecular formula of C_14_H_17_N_3_O_2_, with a yield 86% and yellow powder and melting point of 80–85°C. It has UV-Visible λ_max_ (methanol) of 383 nm, IR [ʋ cm^−1^, KBr (pellet)]: 3368 ν(O-H), 3275 ν(N-H), 1639 ν(imine C=N). Composition: Calc. for C_14_H_17_N_3_O_2_; C 64.85; H 6.61; N 16.20; O 12.34%. Found C 64.71; H 6.65; N 16.08 and O 12.56%. ^1^H NMR (400 MHz, DMSO-d6): *δ*_H_ 3.65 (8H, *d*, H-11, H-12, H-14 and H-15), 4.72 (1H, *s*, OH), 4.92 (1H, *s*, OH), 7.19 (1H, *t*, *J* = 7.25Hz, H-6), 7.55 (2H, *m*, H-5, H-8), 7.72 (1H, *d*, *J* = 8.36 Hz, H-7), 8.21 (1H, *s*, H-4), 8.5(1H, *s*, H-9), and 9.55 (1H, *s*, NH); ^13^C NMR (400 MHz, DMSO-d6): *δ*_C_ 43.4 (C-14), 60.5 (C-12), 61.2 (C-15), 63.7 (C-11), 117.2 (C-3), 121.9 (C-8), 122.4 (C-4a), 125.7 (C-5), 128.9 (C-6), 131.5 (C-7), 143.0 (C-4), 148.3 (C-8a), 155.4 (C-2), and 163.8 (C-9); DEPT-135 δC 43.4 (C-14 negative), 60.5 (C-12 negative), 61.2 (C-15 negative), 63.7 negative (C-11), 121.9 (C-8), 125.7 (C-5), 128.9 (C-6), 131.5 (C-7), 143.0 (C-4) and 163.8 (C-9). Composition: Calc. for C_14_H_17_N_3_O_2_; C 64.85; H 6.61; N 16.20; O 12.34%. Found C 64.71; H 6.65; N 16.08 and O 12.56% (Supplementary Figures S1–S3, S4B).

### 2.4 Synthesis of the metal complexes

**SCHEME 1:**
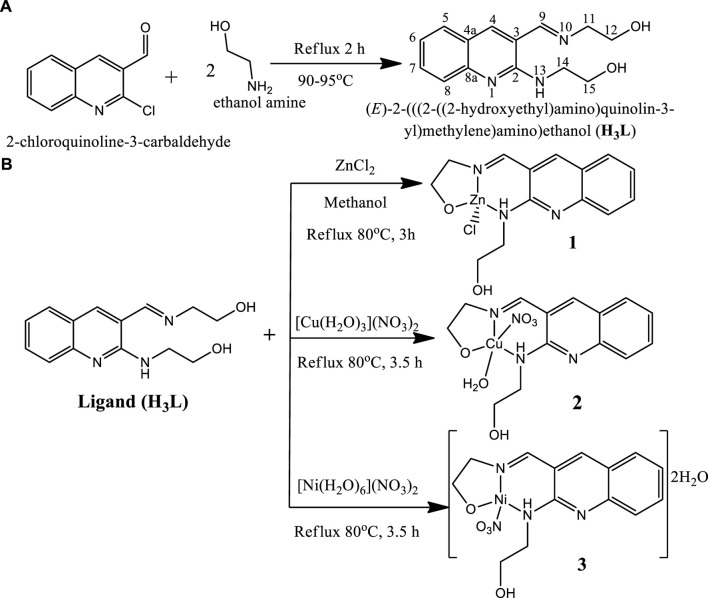
Proposed synthetic reactions of **(A)** ligand (H_3_L) and **(B)** its metal complexes.

A drop of triethylamine was added to stirred solution of the ligand (0.25 g, 0.96 mmol,) in methanol (10 ml). After 30 min of stirring, a solution (0.96 mmol) of ZnCl_2_ (0.13 g), Cu(NO_3_)_2_·3H_2_O (0.232 g), and Ni(NO_3_)_2_.6H_2_O (0.279 g) in methanol (10 ml,) was added dropwise to this solution separately in different flasks (Damena et al., 2022a; Damena et al., 2022b). The mixture was refluxed for 3 h and 3.5 h at 80°C, respectively for the Zn(II), Cu(II) and Ni(II) complexes. Progress and completion of the reaction was monitored with thin layer chromatography. After completion, the reaction mixture was cool down at room temperature and the precipitated product was filtered off, washed with ice cold methanol and dried at room temperature based on reported procedures (Zou et al., 2017; Ramachandran et al., 2018; Naz et al., 2020; Halevas et al., 2021; Sumalatha et al., 2021; Damena et al., 2022a). Finally, light yellow, deep green and reddish brown powder product was obtained respectively (Supplementary Figure S5). The proposed reaction mechanisms are presented in Scheme 1.

#### 2.4.1 Complex 1

Complex **1** was found to be light yellow polycrystalline powder with molecular formula [Zn(H_2_L)Cl], yield 63%, melting point 225–230°C. It is soluble in polar solvents. Compositions calculated for **1** are C 46.82, H 4.49, N 11.70, O 8.91, Cl 9.87 and Zn 18.21%; found: C 46.85, H 4.36, N 11.80, O 8.55, Cl 10.25 and Zn 18.19%. FTIR (ʋ cm^−1^, KBr (pellet)): 1657 ν(Imin C=N), 1034 ν(C-O), 524 ν(Zn-O), and 460 ν(Zn-N). UV-Vis (methanol, nm): 231 (π→π*), 258 (π→π*), 300 (n→π*) and 380 (n→π*).

#### 2.4.2 Complex 2

Complex **2** was found to be deep green polycrystalline powder with molecular formula [Cu(H_2_L)(H_2_O)(NO_3_)], yield 61.5%, melting point 195–200°C. It is soluble in polar solvents. Compositions calculated for **2** are C 41.84, H 4.51, N 13.94, O 23.89 and Cu 15.81%; found: C 41.62, H 4.90, N 13.80, O 24.15, and Cu 15.53%. FTIR (ʋ cm^−1^, KBr (pellet)): 1652 ν(Imin C=N), 1059 ν(C-O), 626 ν(Cu-O), and 474 ν(Cu-N). UV-Vis (methanol, nm): 235 (π→π*), 267 (n→π*), 317 (n→π*) and 406 (LMCT).

#### 2.4.3 Complex 3

Complex **3** was found to be brownish purple polycrystalline powder with molecular formula [Ni(H_2_L)(NO_3_)].2H_2_O, yield 66%, melting point 115–120°C. It is soluble in polar solvents. Compositions calculated for **3** are C 40.52, H 4.86, N 13.50, O 26.99 and Ni 14.14%; found: C 40.86, H 4.95, N 13.35, O 26.90 and Ni 13.94%. FTIR (ʋ cm^−1^, KBr (pellet)): 1650 ν(Imin C=N), 1037 ν(C-O), 534 ν(Ni-O), and 462 ν(Ni-N). UV-Vis (methanol, nm): 229 (π→-π*), 259 (π→π*), 302 (n→π*) and 401 (LMCT).

### 2.5 Formation constants and thermodynamic parameters

Zn(II) chloride, Cu(II) nitrate trihydrate, and Ni(II) nitrate hexahydrate standard solutions were pipetted into ten 50 ml volumetric flasks (0, 1, 2,... 10 ml), and aliquots of a similar standard solution of the precursor ligand were added (10, 9, 8... 0 ml). All absorbance were recorded at λ_max_ 380, 406, and 401 nm, respectively, for complexes **1**, **2** and **3**, at temperatures of 25, 30, 37 and 40°C. A drop of triethylamine was used to keep the pH of the mixture constant. The metal ion and free ligand mole fractions were changed between 0 and 1 for stoichiometric measurement. From this, the absorbance of the solutions was plotted with these mole fractions, and “n”, the average number of bound ligand, was obtained from the plot, where *X*_max_ was calculated using Eq. 1 (Ahmad et al., 2012; Shalaby and Mohamed, 2020).

$$n=\frac{X_{\max}}{1-X_{\max}}$$ (1)

Additionally, spectroscopic analysis was used to estimate the complexes’ formation constants (Supplementary Eq. S1). The changes in enthalpy and entropy (Δ*H* and Δ*S*) were derived from the slope and intercept of the lnK vs. 1/T (van’t Hoff) plot, respectively, in order to evaluate the thermodynamic parameters (Δ*G*, Δ*H*, and Δ*S*) appropriately (Ahmad et al., 2012; Damena et al., 2014; Shalaby and Mohamed, 2020). Eqs 2, 3 were combined to get the reactions’ Gibbs free energy (Δ*G*).

ΔG=ΔH−TΔS (2)

ΔG=−RT ln⁡K (3)

### 2.6 Antibacterial activity

Antibacterial activities of the newly synthesized Zn(II), Cu(II) and Ni(II) complexes (**1**–**3**) were evaluated using disc diffusion method against two Gram-positive (*Staphylococcus aureus*, ATCC25923 and *Streptococcus pyogenes*, ATCC19615) and two Gram-negative (*Escherichia coli*, ATCC 25922, and *Pseudomonas aeruginosa*, ATCC 27853) bacteria by following previously reported media preparation methods (Damena et al., 2022a; Damena et al., 2022b). The antibacterial activities were recorded for two sample concentrations (150 and 300 *μ*g/ml) in DMSO. Ciprofloxacin and DMSO were used as a positive and negative control, respectively. The plates were incubated at 37°C for 48 h, and then the bacterial growth data were evaluated by measuring the inhibition zones according to literature (Kargar et al., 2021; Damena et al., 2022a; Damena et al., 2022b). All experiments were performed in triplicate, and the mean of the triplicates was reported. The bacterial activities of the synthesized complexes were confirmed by calculating activity index (AI) (El-Gammal et al., 2021), Eq. 4.

$$\%\;Activity\;index\;\left(AI\right)=\frac{Mean\;inhibition\;zone\;of\;compounds}{Mean\;inhibition\;zone\;of\;standard}\times 100$$ (4)

### 2.7 Antioxidant activity

The radical scavenging activity study of the ligand and its Zn(II), Cu(II) and Ni(II) complexes were determined using a DPPH assay based on the reported studies (Fetoh et al., 2018; Kumar et al., 2020; Sumalatha et al., 2021). Accordingly, various samples (5, 10, 25, 40, 55, 70, 85, 100, and 115 μg/ml) and the assay concentration (40 ppm) was prepared, in which 2 ml of the assay solution was mixed with 2 ml of each of the titled sample compounds. The control was prepared from 2 ml of the assay (DPPH) solution and 2 ml of solvent (methanol). The vigorously shaken resulting mixtures were put into dark incubator (Labfreez: TSI-200) at 37°C for 30 min and absorbance was recorded at 517 nm in triplicates. The percentage of radical scavenging was determined from average absorbance using Eq. 5 based on reported studies (Kumar et al., 2020; Halevas et al., 2021):

$${DPPH}_{radical\;scavenging\;activity\;\left(\%\right)}=\left[\frac{\left(A_{i}-A_{S}\right)}{A_{i}}\right]\times 100\%$$ (5)

where A_i_ and A_S_ are the absorbance of the control and sample with control solution, respectively. Finally, the half-maximal inhibitory concentration (IC_50_) was determined from the slope and intercept of the plot of percent radical scavenging activity vs. concentration.

### 2.8 Computational methods

#### 2.8.1 Drug likeness and ADME prediction

Absorption, Distribution, Metabolism, and Excretion (ADME) prediction was performed with SwissADME webtool to understand the safety and efficacy of the metal complexes as drug candidates. The Swiss Institute Bioinformatics (SIB) webtool (SwissADME) was used to convert the two dimensional structure into its simplified molecular input line entry system (SMILES) and then to estimate the *in silico* pharmacokinetic properties (Daina et al., 2017). In line with the experiment, ciprofloxacin was used as a positive control.

#### 2.8.2 DFT calculations

Geometry optimizations of the ligand (**H**_**3**_ **L**) and its metal complexes (**1**–**3**) were performed using the Gaussian 16 program package (Frisch et al., 2016) and the results were visualized using GaussView 06 and Chemcraft. The density functional theory (DFT) and time dependent DFT (TD-DFT) calculations were performed using the B3LYP hybrid functional (Lee et al., 1988; Becke, 1993; Stephens et al., 1994) together with 6–311++G (d,p) basis set (Krishnan et al., 1980) for the light atoms and LanL2DZ basis sets for the metal atoms to account for relativistic effects. Grimme’s dispersion correction (Grimme, 2004) was employed to treat non-bonding interactions during the calculations. Such combination of functional and basis sets has been used in our previous studies (Demissie and Hansen, 2016; Bitew et al., 2021; Demissie et al., 2021; Damena et al., 2022a; Damena et al., 2022b). The polarizable continuum model in its integral equation formalism (IEF-PCM) (Tomasi et al., 2005) was used with methanol solvent to rectify the solvent effects in order to match the experimental conditions. Vibrational frequency calculations were done on the optimized geometries at the same theoretical level, which proved that there were no imaginary vibrational frequencies present and that they were true minima. The wave function distributions of the lowest unoccupied molecular orbital (LUMO), the highest occupied molecular orbital (HOMO), and their Eigen values were estimated. Quantum chemical descriptors such as band gap energy (*E*_*g*_ = *E*_LUMO_–*E*_HOMO_), electronegativity (*χ* = -½ (*E*_HOMO_ + *E*_LUMO_)), electronic chemical potential (*μ* = ½ (*E*_HOMO_ + *E*_LUMO_) = −*χ*), global chemical hardness (*η* = ½ (*E*_LUMO_–*E*_HOMO_)), global softness (*σ* = 1/2*η*), global electrophilicity index (*ω* = *μ* ^*2*^ */2η*), nucleophilicity index (*Nu* = 1/*ω*)*,* and dipole moment were calculated and analyzed at the same level of theory (Ismael et al., 2020).

#### 2.8.3 Molecular docking analysis

Using AutoDock 4.2.6 (MGL tools 1.5.7) and a standard methodology, the molecular docking experiments of the free ligand and its metal complexes (**1**–**3**) were carried out (Allouche, 2012) against the active sites of the proteins of *E. coli* DNA gyrase B (PDB ID: 6F86) and *P. aeruginos*a LasR. (PDB ID: 2UV0). With a grid point spacing of 0.375, the grid box was built using 58, 58, and 40 points that pointed in the x, y, and z directions, respectively. The grid box’s center was 14.527, 56.689, and 5.122. The Scripps Institute website (Allouche, 2012) was utilized to download the atom properties for the metal centers (Grimme, 2004). Using AutoDock scoring routines, hundred alternative conformations for the ligand and its metal complexes were produced and sorted by binding energies. The post-docking evaluations were conducted using PyMOL and AutoDock Tools. The conformations with the lowest free binding energies were chosen to analyze and visualize the interactions between the compounds and the target receptor using PyMOL and Discovery Studio (Rigsby and Parker, 2016).

### 2.9 Statistical analysis

The bacterial activities evaluation data with triplicate measurements were determined as mean ± standard deviation, in which GraphPad Prism version 5.00 was used for the analysis (GraphPad Software, California, United States) (Mogana et al., 2020). Groups were analyzed for significant differences using analysis of variance (ANOVA) test for correlation with significance (*p* < 0.05) (Supplementary Table S1).

## 3 Results and discussion

### 3.1 Molar conductance

The molar conductance of complexes **1** – **3** were found to be 5.21, 18.57, and 15.07 Ω^−1^mol^−1^cm^2^, respectively (Supplementary Table S2), indicating non-electrolytic nature of the complexes (Chandra and Kumar, 2005; Ismael et al., 2020). This is mainly due to the fact that the metal cations receive electrons from the ligand to make a net charge balance of zero. The molar conductance results can also provide information about the proposed structures of the complexes. Hence, the chloride test of Zn(II) complex was performed using a drop of silver nitrate solution. The absence of white precipitate confirmed that chloride ion was coordinated to central metal ion in the inner sphere of the Zn(II) complex, hence the proposed formula of this complex is represented with [Zn(H_2_L)(Cl)] and this in line with reported studies (Ismael et al., 2020; Damena et al., 2022b). Although it was discovered that most metal complexes exhibited a non-electrolytic nature, copper (II) and nickel (II) complexes have substantially higher molar conductance than zinc (II) complexes. This could be as a result of the Zn(II) complex’s low solubility, which impacts its ion mobility and molar conductance. A previous study suggested that the low molar conductivity of the Zn(II) complex may potentially be due to the absence of anions beyond the coordination sphere (Condé et al., 2022). Furthermore, this analysis was found to be in line with the DFT optimized geometries (*vide infra*).

### 3.2 Formation constants and thermodynamic parameters

The Job’s plot for mole fraction of the ligand and the metal ions are presented in Supplementary Figure S6. It was found that the maximum point was recorded at a mole fraction (X) of 0.5, evidencing the synthesized complexes have 1:1 [M:H_3_L] ratio (Supplementary Table S3). Similar results were obtained at temperature elevations up to 40°C (Ahmad et al., 2012; Shalaby and Mohamed, 2020). The thermodynamic parameters were determined from the plot of lnK vs. 1/T (Supplementary Figure S7). The negative values obtained for the change in Gibbs free energy and enthalpy showed that the complexes are thermally stable up to 40°C (Table 1). The metal-ligand interactions showed spontaneity and exothermic nature. This is due to larger negative values of Gibbs free energy (Δ*G*) and lower negative values of enthalpy change (Δ*H*) of the chemical reactions. In other cases, the complex formations are entropically favored due to positive values of change in entropy (Δ*S*) (Ahmad et al., 2012; Shalaby and Mohamed, 2020; Damena et al., 2022b). Overall, the formation constants of the complexes remain constant with increase in temperature, inferring that the complexes are stable up to 40°C (Supplementary Table S3). This is in line with the thermal analysis study of all the three complexes in which no mass loss was observed up to 100°C (*vide infra*).

**TABLE 1:** Thermodynamic parameters of the synthesised complexes.

Complexes	**1**				**2**				**3**			
Temp. (^o^C)	25	30	37	40	25	30	37	40	25	30	37	40
Lnk	14.7	14.7	14.7	14.7	14.9	14.9	14.9	14.9	12.9	12.9	12.9	12.9
−Δ*G* (kJ/mol)	36.4	37.0	37.8	38.2	36.9	37.5	38.4	38.8	31.8	32.4	33.1	33.4
−Δ*H* (kJ/mol)	0.4				0.8				0.1			
Δ*S* (J/mol)	122.5				119.3				106.7			

### 3.3 FTIR analysis

After the successful synthesis of the complexes, the presence and disappearance of characteristic functional groups in the targeted compounds were identified from the FTIR spectral data (Supplementary Figures S8, S9). Accordingly, the spectra showed strong stretching band at 1639 cm^−1^ which was assigned for imine ν(C=N) group (Im) of the ligand (Supplementary Figure S8A), but in the case of all complexes **1**–**3**, this spectral band shifted towards higher frequency range of 1647–1688 cm^−1^ (Supplementary Table S4, Supplementary Figures S8B, S9C,D). This confirms the involvement of the donor nitrogen atom of the imine group ν(C=N) coordinated with the metal ions (Senthil et al., 2012; El-Sonbati et al., 2019). Similar to this, the loss of the free ligand’s ν(O-H) stretching frequency at 3368 cm^−1^ provides proof that the hydroxyl group’s oxygen atom participated in the creation of metal-oxygen bonds (Ali et al., 2019). In addition, the characteristic ν(N-H) stretching frequency of the ligand at 3275 cm^−1^ has shifted to 3294, 3168, and 3197 cm^−1^ for Zn(II), Cu(II) and Ni(II) complexes, respectively (Supplementary Table S4). This also indicates the participation of the amine group in the formation of a dative bond during the complex formation process (Ali et al., 2019; Indira et al., 2019).

The IR spectral data of Ni(II) and Cu(II) complexes have weak and broad spectral bands of stretching frequencies in the range 3673–3373 and 3664–3334 cm^−1^, respectively (Supplementary Table S4). This could be attributed to the vibration of the water molecules that might be present as lattice water and coordination water, respectively. These are newly emerged medium stretching vibration bands at 524 cm^−1^ ν(Zn-O) and 460 cm^−1^ ν(Zn-N) for the Zn(II) complex; 626 cm^−1^ ν(Cu-O) and 474 cm^−1^ ν(Cu-N) for the Cu(II) complex; and 534 cm^−1^ ν(Ni-O), 462 cm^−1^ ν(Ni-N) for the Ni(II) complex. The DFT calculated frequencies are in close agreement with the corresponding experimental results (Supplementary Table S4). The visualization of the DFT calculated frequencies confirmed that all the three metal ions were involved in binding to the ONN donor atoms of the ligand during complex formation (Abd El-Halim et al., 2017; Ali et al., 2019; Indira et al., 2019). The presence of nitrate in both Cu(II) and Ni(II) complexes were confirmed by the strong and broad band IR peaks at 1380 and 1351 cm^−1^, respectively, in agreement with previously reported studies for related complexes (Ali et al., 2019; Condé et al., 2022). Weak O-H bending vibration was observed in the range of 1437–1460 cm^−1^ in all the three complexes, confirming the appearance of free bending hydroxyl (O-H) groups. Overall, the close agreement between the DFT calculated IR frequencies and the corresponding experimental results (Supplementary Table S4) further confirmed the analysis.

### 3.4 UV-visible spectroscopy

**FIGURE 1:**
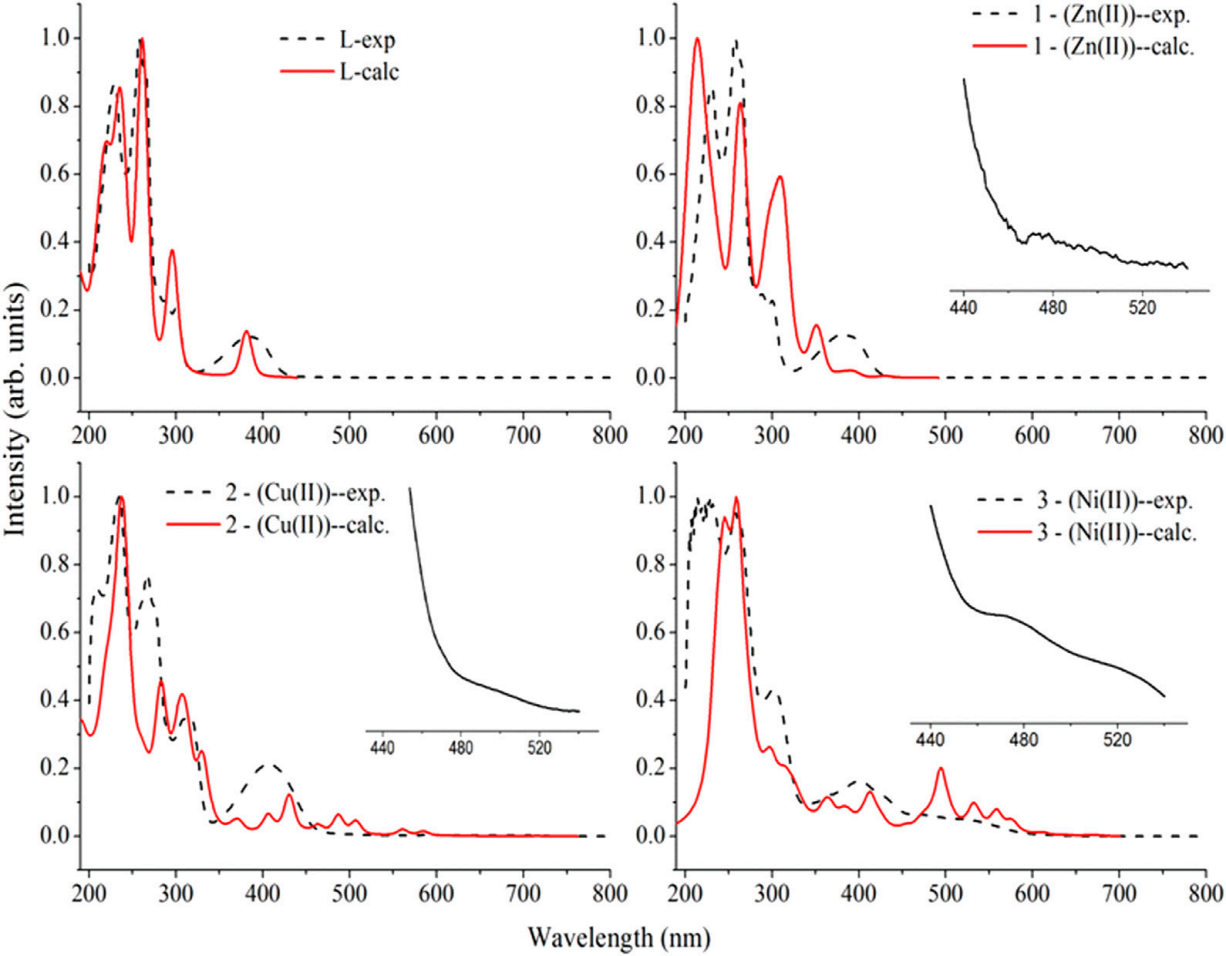
Comparison of the experimental absorption wavelengths with the corresponding TD-B3LYP-GD3/6–311++G (d,p)/LanL2DZ/IEF-PCM/methanol calculated results of the ligand (**H**_**3**_ **L**), the Zn(II) (**1**), Cu(II) (**2**), and Ni(II) (**3**) complexes. The calculated absorption maxima were red shifted by 20 nm for better comparison with the experimental results. Insets are experimental plots for the range between 440 and 540 nm.

The electronic spectra of the free ligand and the associated Zn(II), Cu(II), and Ni(II) complexes revealed that the ligand absorption bands are primarily caused by π→π* and n→π* transitions in UV-Visible spectral data between 200 and 800 nm. The peak for Cu(II) and Ni(II) complexes are red shifted, whereas very small blue shift was observed for the Zn(II) complex. The free ligand showed absorption peaks at 231, 258 nm (π→π*) and 300, 383 nm (n→π*) (Table 2). The red shifts observed for the Cu(II) and Ni(II) complexes at 406 and 401 nm, respectively, are mainly due to ligand-based transitions and ligand to metal charge transfer (LMCT) (Sathisha et al., 2008; Güveli et al., 2014; Halevas et al., 2021). This is because the ligand has lone pair electrons and both Cu(II) and Ni(II) complexes have vacant d-orbitals (Dalal M Charge Transfer Spectra; Sallam et al., 2011). The small band gap energy (3.272 eV) of the Cu(II) complex resulted LMCT from the quinoline ring π-orbitals to the metal d-orbitals. Moreover, as it can be seen from the insets of the absorption plots presented in Figure 1, there is a very weak d→d transition around 480 nm in the case of the Ni(II) complex. Due to the predominance of inter-ligand electron transfers, the Zn(II) complex underwent only minor modification. This might be as a result of Zn(II) having a d^10^ electron configuration, which prevents it from taking part in the d→d transition (Ekennia et al., 2015). This is clearly observed from the inset for the experimental absorption plot of the Zn(II) complex. The analysis was supported using the TD-DFT calculated absorption spectra of the complexes. The calculated spectra also showed the same trends and are in very good agreement with the corresponding experimental results (Figure 1).

**TABLE 2:** Electronic spectra of the ligand and the corresponding Zn(II), Cu(II) and Ni(II) complexes.

	Absorption (nm)	Transition
H_3_L	231, 258, 300, 383	(π→π*), (n→π*) and (n→π*)
1	231, 258, 300, 380	(π→π*), (n→π*) and (n→π*)
2	235, 267, 317, 406	(π→π*), (n→π*) and LMCT
3	229, 259, 302, 401	(π→π*), (n→π*) and LMCT

**FIGURE 2:**
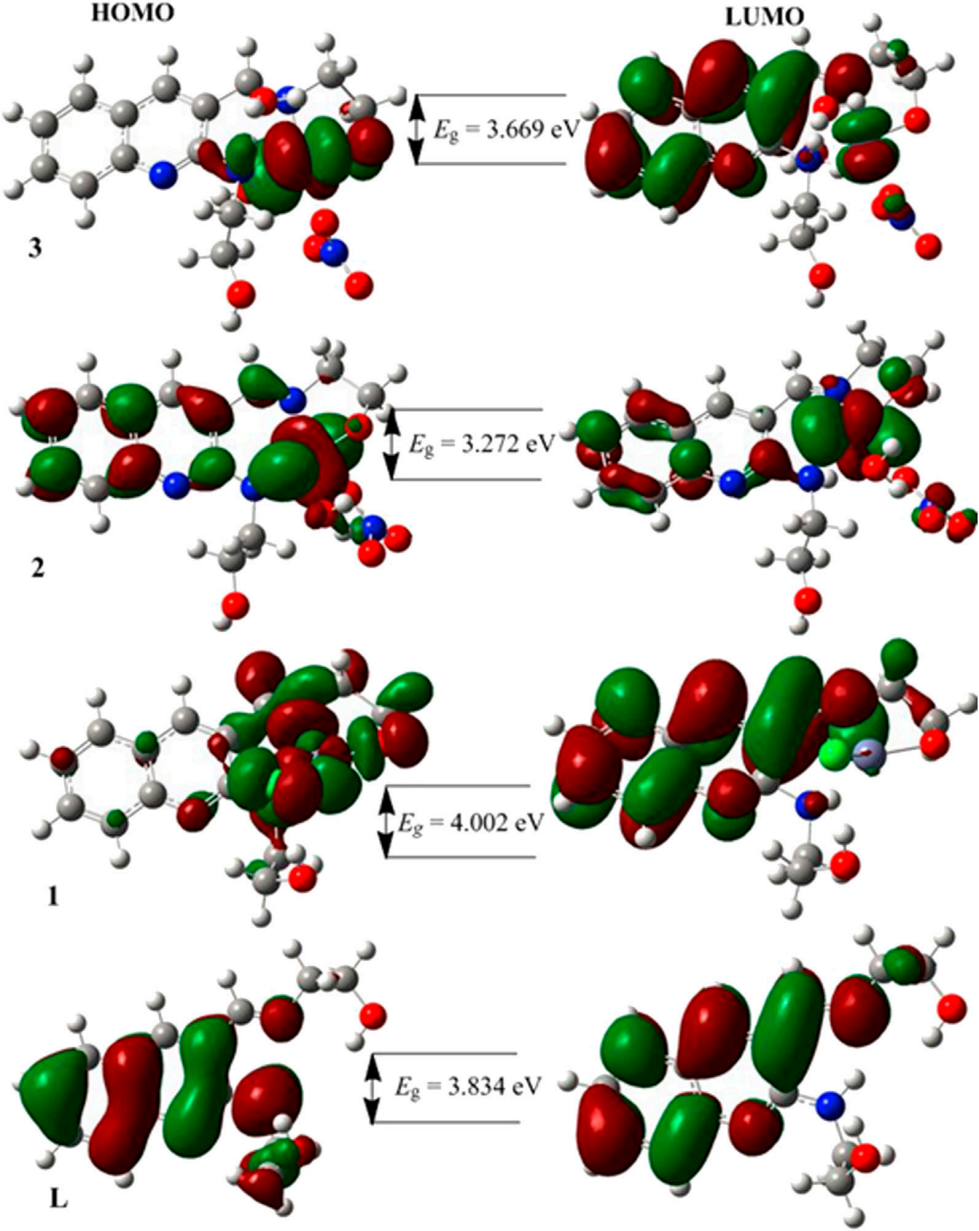
The frontier molecular orbitals (HOMO and LUMO) of the ligand and its metal complexes **1**–**3**.

The characteristics strong bands that appeared between 259–383 nm in all the three metal complexes were attributed to intra-ligand (C-N or C-O group) electronic transitions, in line with literature (Ilhan et al., 2014; Mahmoud et al., 2015; Abd El-Halim et al., 2017). In addition, the strong absorption bands of all the three metal complexes in the UV region (229–258 nm) could be assigned to the *N*-quinoline ring (ligand-based π→π* transitions). The absorption bands above 400 nm could be assigned to the LMCT of Cu(II) and Ni(II) complexes (Table 2) in agreement with reported studies (Ilhan et al., 2014; Mahmoud et al., 2015; Abd El-Halim et al., 2017). Overall, the analysis of the electronic spectra revealed that d→d transition was dominated by LMCT phenomenon and hence the resulted colors of the complexes (Abd El-Halim et al., 2017). Moreover, the electronic transitions were analyzed based on the frontier molecular orbital (FMO) plots presented in Figure 2. The HOMO and LUMO of the **H**_**3**_ **L** reside on the quinoline ring, confirming the presence of π→π* electron transition. It is also observed that the electron densities of the HOMO reside on the amine part of the molecule and the LUMO reside on its imine part. This is due to the fact that the amine and imine part of the ligand are in the same plane making it suitable for metal coordination (Hamdani et al., 2020). The HOMO and LUMO of compounds **1** and **3** are delocalized over the metal centers (Zn and Ni) and the quinoline ring, respectively, inferring the presence of electron transition from metal to the ligand orbital systems for Zn(II) and weak d→d transition for Ni(II) complex. Since the d-orbitals of Zn(II) are fully occupied, there is no d→d transition in **1** (see inset of Figure 1).

### 3.5 Fluorescence study

Fluorescence study was performed at room temperature. The spectra of the ligand (**H**_**3**_ **L**) and its complexes **1**–**3** showed emission bands at 526, 608, 471, and 511 nm, respectively (Table 3 and Supplementary Figure S10). Previous reports indicated that the photoluminescence properties of Zn(II) complexes are mainly due to intraligand emissions because of the presence of d^10^ electron configuration (Aslkhademi et al., 2019; Szemik-Hojniak et al., 2020; Halevas et al., 2021). In the case of Cu(II) and Ni(II) complexes, the emission intensity enhancement could be mainly due to LMCT (Halevas et al., 2021; Zheng et al., 2022). The complexes showed hyperchromic (intense), hypsochromic (blue) shifts, and intense fluorescent bands than the ligand (Supplementary Figure S10). This could be due to an increase in the conformational rigidity of the ligand upon metal coordination. Hence, the metal complexes could potentially be used for photochemical applications (Mandewale et al., 2019; Szemik-Hojniak et al., 2020).

**TABLE 3:** The emission data of free ligand with its Zn(II), Cu(II) and Ni(II) complexes.

	Absorption λ_max_ (Intensity)	Emission λ_max_ (Intensity)
H_3_L	383 (0.16)	526 (22.61)
1	380 (0.35)	608 (96.37)
2	406 (0.32)	471 (35.22)
3	401 (0.46)	511 (44.04)

### 3.6 Powder X-ray diffraction study

**FIGURE 3:**
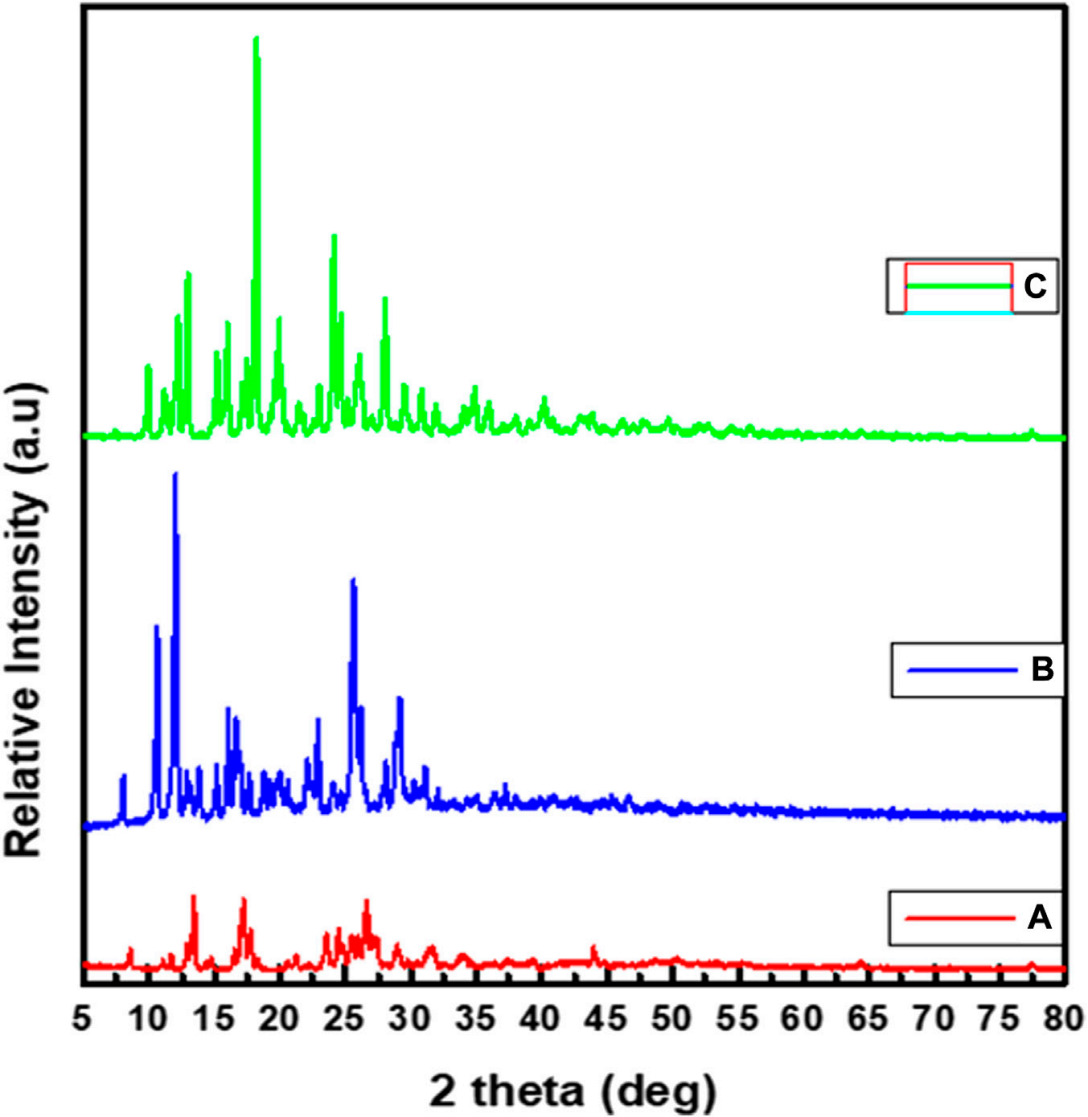
Powder XRD spectral data of: **(A)** Zn(II), **(B)** Cu(II) and **(C)** Ni(II) complexes.

The powder X-ray diffraction (PXRD) patterns of the three synthesized complexes (**1**–**3)** have polycrystalline characteristic peaks (Figures 3A–C), in line with the previously reported studies (Nagesh et al., 2015; Morgan et al., 2017; Ramachandran et al., 2018; Sumalatha et al., 2021). The average crystallite size (D) evaluated from the XRD pattern according to Debye–Scherrer equation as reported in previous studies (Morgan et al., 2017; El-Sonbati et al., 2019; Sumalatha et al., 2021).

$$D=\frac{K\lambda }{\beta \cos\theta }$$ (6)

The equation uses the reference peak width at angle θ, where λ is the wavelength of X-ray radiation (1.5406 Å), *K* is Scherrer constant (0.9) and β is the width at half maximum of the reference diffraction peak measured in degrees (El-Sonbati et al., 2019). The average crystallite sizes of complexes **1**–**3** were found to be 27.86, 33.54 and 37.40 nm, respectively, in good agreement with previous reports for related complexes (Morgan et al., 2017; Ramachandran et al., 2018). This calculation was done by taking average size of the three major peaks. In addition to this the main reflections were defined based on maxima at 2θ that correspond to d values in which the inter-planar spacing (d) was calculated by using Bragg’s equation, λ n = 2dsinθ. The calculated inter-planar d-spacing together with relative intensities with respect to most intense peak have been recorded and then h^2^+k^2^+l^2^ values were determined and based on this value, absence of forbidden numbers (7, 15, 23, 71) tell us that cubic or orthorhombic system (translational symmetry) based on the unit cell calculated values. According, the Zn(II) complex showed absence of the forbidden numbers (7, 15, 23, 71) (Supplementary Table S5), indicating that it could be belong to the face centered cubic system with a, b and c values of 7.28, 7.24, and 6.83 Å, respectively (Morgan et al., 2017). Similarly, the calculated lattice parameters (a, b and c) for the Cu(II) complex were found to be 21.80, 3.86, 8.61, respectively. It was observed that the absence of forbidden numbers (7, 15, 23, 71) (Supplementary Table S5) shows that the Cu(II) complex could belong to orthorhombic systems (Nagesh et al., 2015; Ramachandran et al., 2018). Similar calculations were done for the Ni(II) complex and the results showed that the Ni(II) complex could also belong to orthorhombic systems. Hence, the calculated unit cell parameters for Ni(II) complex (a, b and c) were found to be 13.61, 7.47 and 7.02 Å, respectively (Szemik-Hojniak et al., 2020), which is in agreement with previously reported studies for related complexes (Refat et al., 2013; Nagesh et al., 2015; Morgan et al., 2017; El-Metwaly et al., 2019; Ramesh et al., 2020; Abumelha et al., 2021; Sumalatha et al., 2021).

### 3.7 SEM-EDX study

**FIGURE 4:**
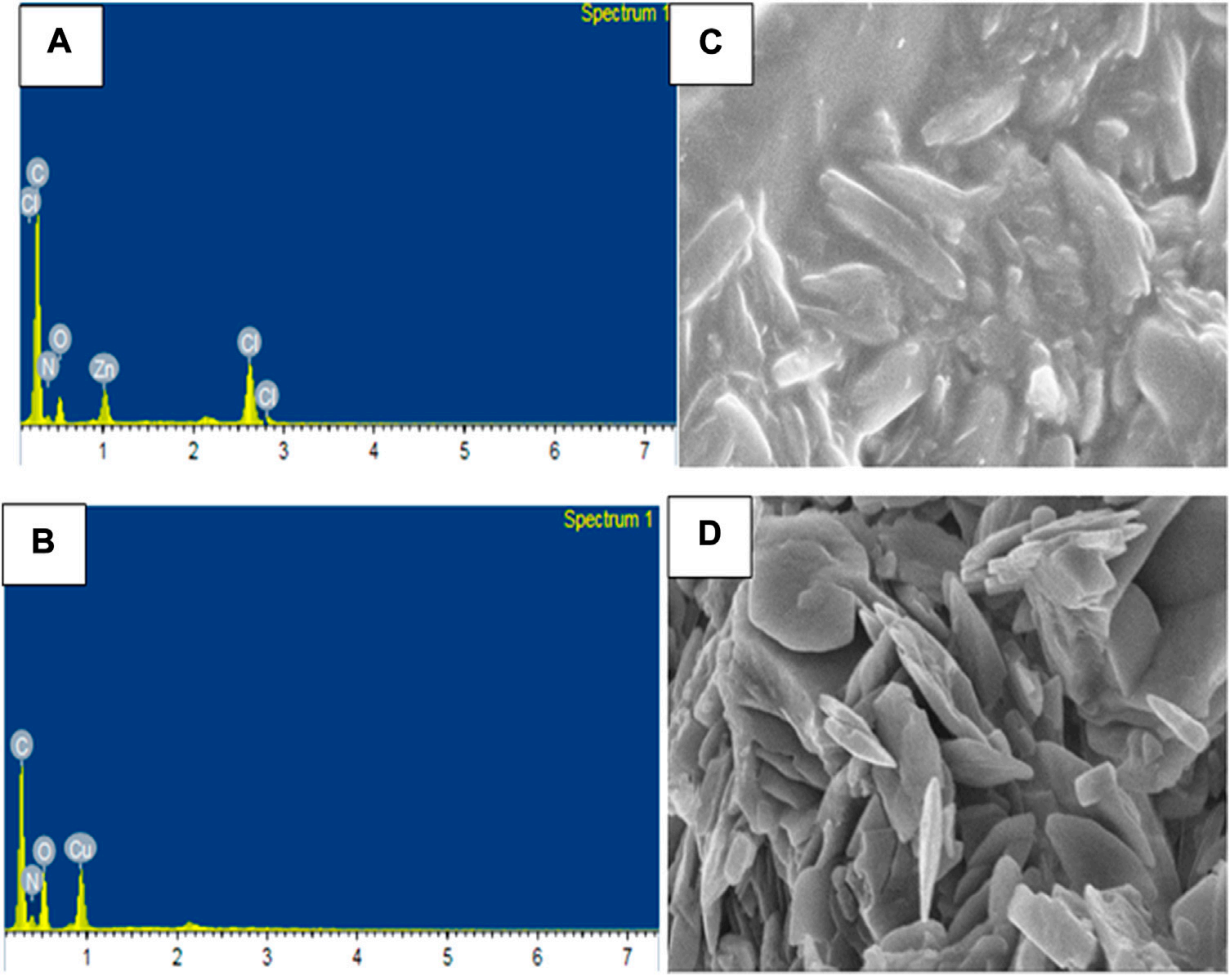
EDX of **(A)** Zn(II) and **(B)** Cu(II), and SEM image of **(C)** Zn(II) and **(D)** Cu(II) complexes.

The compositions of Zn(II), Cu(II) and Ni(II) complexes were analyses from Energy Dispersive X-ray (EDX) analysis, in which the experimental percentage of atoms were found to be very close to the theoretical results (Refat et al., 2013; Sumalatha et al., 2021). In the EDX spectrum of [Zn(H_2_L)Cl] complex, five characteristic signals corresponding to atoms C, O, N, Cl and Zn were observed which confirmed the formation of pure CHZnNOCl (Figure 4A). Similarly, the spectrum of [Cu(H_2_L)(H_2_O)(NO_3_)] showed four signals, which correspond to C, O, N, and Cu atoms, indicating pure CHCuNO (Figure 4B). The [Ni(H_2_L)(NO_3_)].2H_2_O complex showed four signals which correspond to C, O, N, and Ni atoms, and indicate the formation of CHNiNO (Supplementary Figure S4A). The free ligand shows three characteristic signals, which clearly confirms the formation of CHNO compound (Supplementary Figure S4B).

The scanning electron microscopy (SEM) micrographs indicated that the Zn(II) complex shows agglomerates which appear to be stick-like (Figure 4C), whereas the Cu(II) complex shows tiny needles-like agglomerates (Figure 4D), whereas the Ni(II) complex shows flower-like agglomerate structures (Supplementary Figure S4C). From both the SEM and PXRD data, all the three complexes appear to be clear polycrystalline structures in agreement with reported studies (Refat et al., 2013; El-Sonbati et al., 2019; Sumalatha et al., 2021).

### 3.8 Mass spectral study

The mass spectrum of complex **1** showed a parent molecular ion peak at *m/z* 357.05 (found 357.98) which corresponds to the formula [C_14_H_16_ClN_3_O_2_Zn] (Supplementary Figure S11A) with a molecular weight of 359.13 g/mol. This complex has additional peaks at *m*/*z* 322.07 (8.75%) (found 322.05) and 259.63 (54.75%) (found 260.14), attributed to [C_14_H_16_N_3_O_2_Zn]^+^ and [C_14_H_18_N_3_O_2_]^+^ fragments, respectively. Similarly, complex **2** has a parent molecular ion peak at *m/z* 400.08 (found 401.05) which corresponds to the formula [C_14_H_18_CuN_4_O_6_] with a molecular weight of 401.86 g/mol (Supplementary Figure S11B). This complex also showed other peaks at *m*/*z* 322 95 (6.70%) (found 322.06), 260.01 (54.75%) (found 260.14) and 226.13 (14.75%) (found 226.13) corresponding to [C_14_H_17_CuN_3_O_2_]^+^, [C_14_H_18_N_3_O_2_]^+^ and [C_14_H_16_N_3_]^+^ fragments respectively. Finally, complex **3** exhibited a parent molecular ion peak at *m/z* 413.09 (found 414.07) attributed to [C_14_H_20_N_4_NiO_7_] formula with a molecular weight of 415.02 g/mol (Supplementary Figure S11C). As usual this complex showed additional peaks at *m*/*z* 351.09 (10.9%) (found 352.08), 315.09 (30.70%) (found 316.06) and 260.33 (29.9%) (found 260.14) corresponding to [C_14_H_20_N_3_NiO_4_]^+^, [C_14_H_16_N_3_NiO_2_]^+^ and [C_14_H_18_N_3_O_2_]^+^ fragments, respectively. It is important to note that similar observations have been made for other related Ni(II) containing complexes (Bursey, 1974; Tyagi et al., 2015; Morgan et al., 2017; Indira et al., 2019; El-Sonbati et al., 2020). Overall, the analysis of the mass spectra and elemental compositions are in good agreement.

### 3.9 Thermal gravimetric study

**FIGURE 5:**
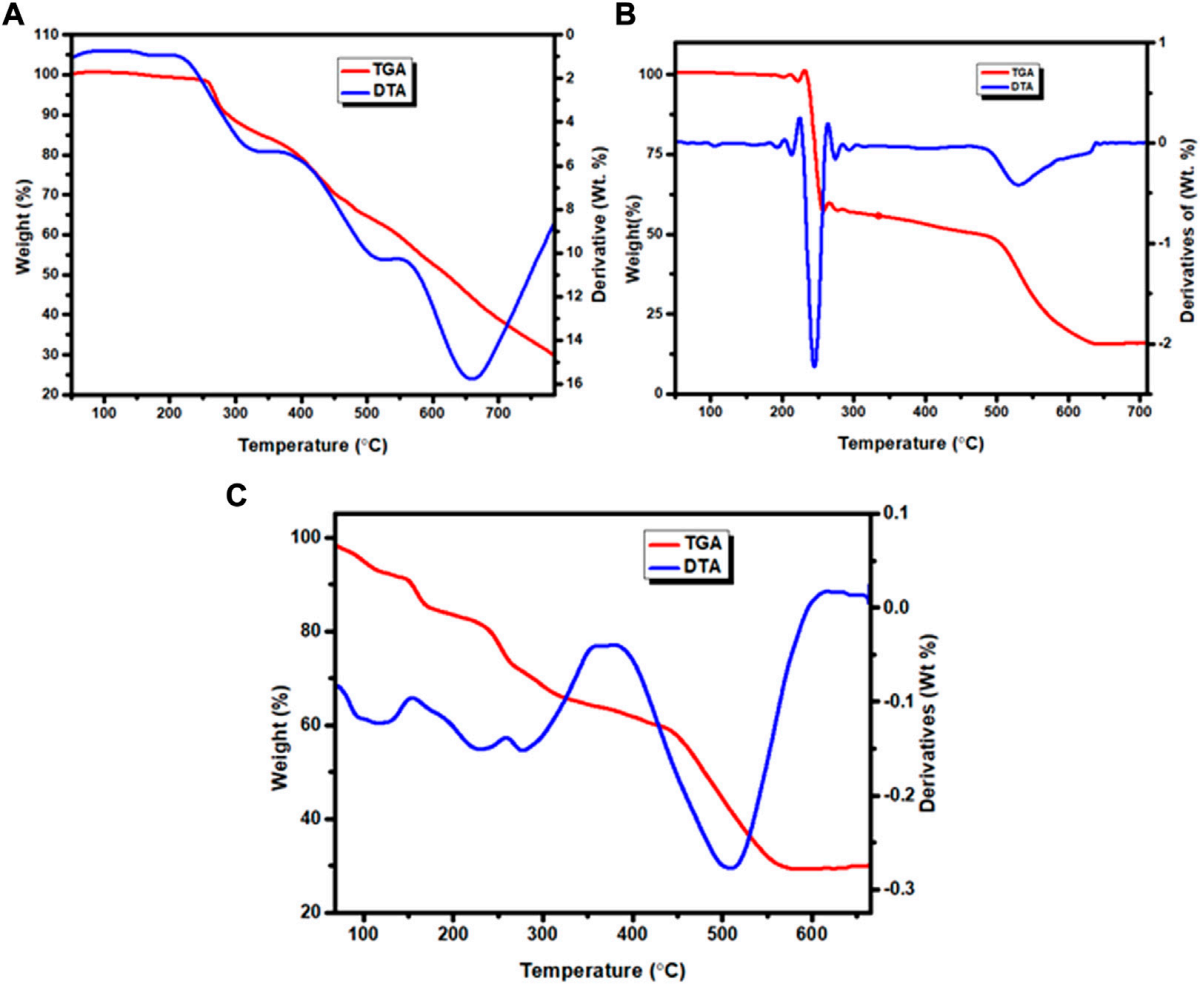
TGA and DTA curves: **(A)** Zn(II), **(B)** Cu(II) and **(C)** Ni(II) complexes.

The weight loss of the complexes was measured at temperature ranges 25–800°C. The results are presented in Figures 5A–C, Supplementary Figures S12A–C and Table 4. The TGA diagram of complex **1** showed three decomposition steps (Figure 5A and Supplementary Figure S12A). The first step of degradation was observed in a temperature range of 255–350°C (DTA_max_ of 325), which indicates a mass loss of 17.80% (calcd. 17.96%) corresponding to the loss of chloroethane (C_2_H_5_Cl) like moiety. The complex was stable up to 200°C indicating the absence of both lattice and a water ligands in the specified complex (Nagesh et al., 2015; Halevas et al., 2021; Sumalatha et al., 2021). The second decomposition observed with a weight loss of 21% (calcd. 21.19%) was attributed to the loss of C_6_H_4_ fragment of the quinoline ring moiety at 360–520°C (DTA_max_ of 515). The third step of degradation was from mass loss of 29.99% (calcd. 30.38%) which corresponds to the loss of C_5_H_5_N_2_O moiety at a temperature range of 535–765°C (DTA_max_ of 655). The actual weight loss occurred from all these steps are 68.79%, which is in good agreement with the calculated result of 69.53%. Gradual degradation was observed up to 765°C and the residue corresponds to zinc oxide (ZnO), 22.52% (calcd. 22.66%), and CHN imine moiety with 7.40% (calcd. 7.53%) of the complex.

**TABLE 4:** TGA and DTA data of Zn(II), Cu(II) and Ni(II) complexes.

	Degradation Temp. (^o^C)	DTA_max_ (°C)	Weight loss (%)		Description
			Obsd.	Calcd.	
**1**	255–350	325	17.80	17.96	Loss due to chloroethane (C_2_H_5_Cl) like moiety
	360–520	515	21.00	21.19	Loss due to C_6_H_4_ species of quinoline ring
	535–765	655	29.99	30.38	Loss due to C_5_H_5_N_2_O moiety of the quinoline ring
**2**	100–225	213	41.69	41.44	Loss of one water molecule, C_3_H_6_N_2_O and nitrate ion moiety
	240–498	245	7.26	7.00	Loss of C_2_H_5_ ethane like moiety
	520–640	530	32.14	31.89	Loss due to C_9_H_6_N moiety of the quinoline ring
**3**	100–155	125	8.65	8.67	Loss of two lattice water molecule
	220–255	229	10.23	10.13	Loss of C_2_H_4_N moiety of imine
	260–361	278	22.44	22.41	Loss of CH_3_O + NO_3_ methanol and nitrate ion moiety
	382–610	508	28.74	28.96	Loss due to C_8_H_9_N, moiety of the quinoline ring

The TGA diagram of complex **2** indicates three decomposition steps (Figure 5B and Supplementary Figure S12B). The first step of degradation was due to mass loss of 41.69% (calcd. 41.69%) corresponding to the elimination of one water molecule, C_3_H_6_N_2_O and nitrate ion moiety at temperature ranges of 100–225°C (DTA_max_ of 213). The second step is due to weight loss of 7.26% (calcd. 7.00%) at temperature ranges 240–498°C (DTA_max_ of 245), which correspond to the elimination of C_2_H_4_ ethane like moiety. The final step occurs at temperature ranges of 520–640°C (DTA_max_ of 530) due to the loss of 32.14% (calcd. 31.89%) related to quinoline ring C_9_H_6_N moiety. The leaving residue of the degradation is CuO, which is 18.91% (calcd. 19.80%) of the complex (Refat et al., 2013; Nagesh et al., 2015; Ali et al., 2019). The overall actual weight loss is 81.09%, which is close to the calculated 80.33%.

The thermal decomposition of complex **3** showed four degradation steps (Figure 5C and Supplementary Figures S12C). The first degradation step was due to mass loss of 8.65% (calcd. 8.67%) at temperature ranges of 100–155°C (DTA_max_ of 125) which correspond to the elimination of two lattice water molecules. The second weight loss of 10.23% (calcd. 10.13%) was observed at a temperature ranges of 220–255°C (DTA_max_ of 229), which is attributed to the loss of C_2_H_4_N imine. The third step showed a mass loss of 22.44% (calcd. 22.41%) at temperature ranges of 260–361°C (DTA_max_ of 278) due to loss of (CH_3_O + NO_3_) methanol and nitrate ion moiety. The final step of this complex is due to mass loss of 28.74% (calcd. 28.96%) at a temperature ranges of 382–610°C (DTA_max_ of 508), which corresponds to the loss of quinoline ring moiety. The leaving residues of nickel oxide (NiO), representing 29.24% (calcd. 27.22%) of the complex. The actual mass loss from all these steps is 70.06%, which is in very good agreement with the calculated result (69.91%).

Overall, the percentage content of elements obtained from both the elemental and TGA analyses are in very good agreement for all the three complexes. The general degradation pattern of the Cu(II) and Zn(II) complexes arise in three stages, while that of Ni(II) complex occurred in four stages. The thermogram of complexes **1**–**3** beyond 765, 640 and 610°C, respectively, showed a straight line, indicating the formation of metal oxides (Ambala and Lincoln, 2020). All the complexes were stable up to 100°C without any weight loss, in line with the spectroscopically evaluated stability constants which do not change up to 40°C (Supplementary Table S5). This is a good indication that the complexes could be potentially important for biological applications. Overall, the results are in very good agreement with the formulae proposed from the analytical data (Scheme 1).

### 3.10 Biological applications

#### 3.10.1 Antibacterial activity

**FIGURE 6:**
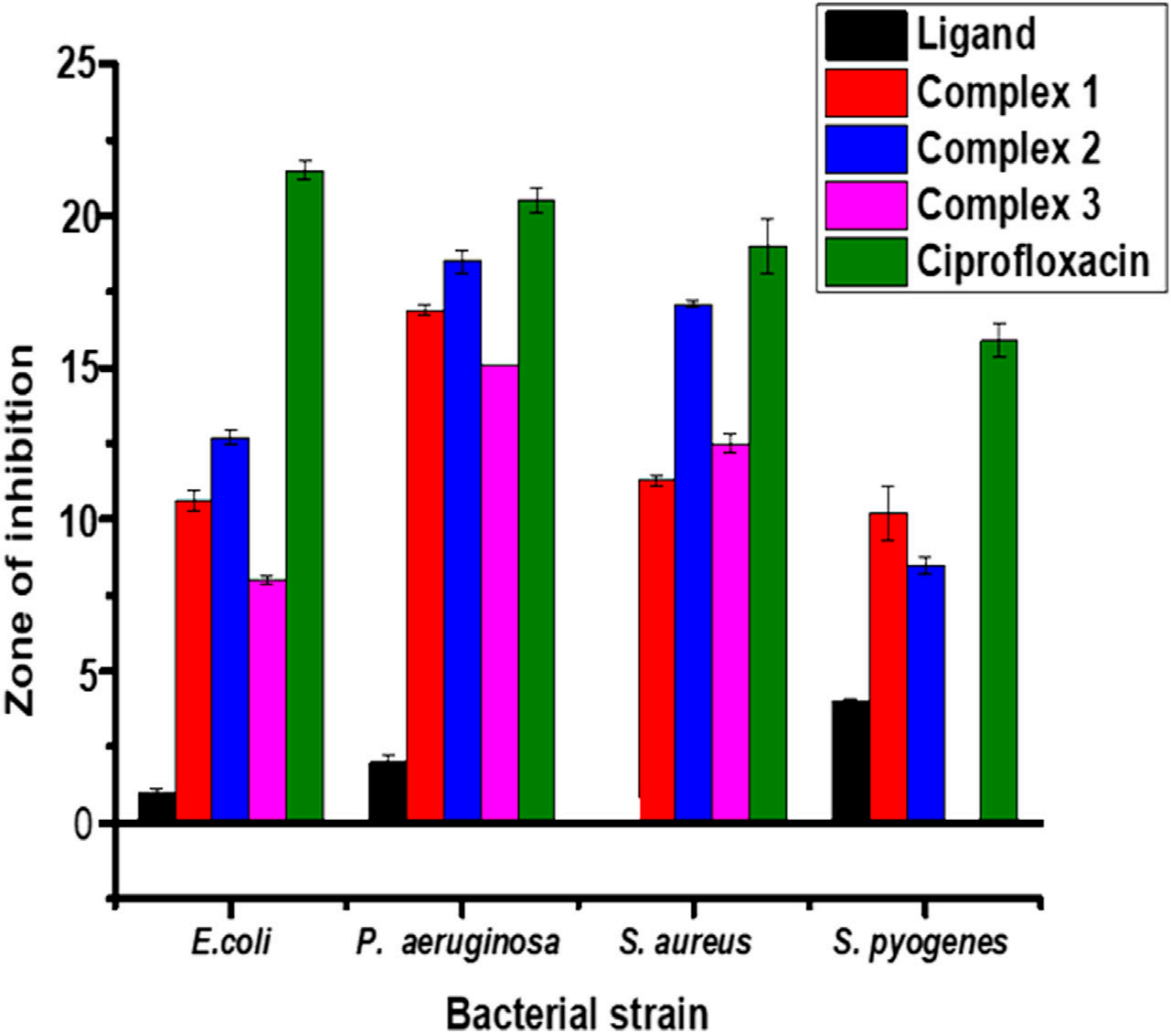
Antibacterial activity of the ligand and its Zn(II), Cu(II) and Ni(II) complexes at 150 μg/ml. Error bars indicate standard deviations.

The results from *in vitro* antibacterial activity study of the ligand and complexes **1**–**3** are presented in Table 5 and Figure 6. The mean inhibition zone (MIZ) of the compounds showed potential antibacterial activity compared to ciprofloxacin. The ligand used in this study was prepared based on the quinoline scaffold. Thus, it is natural to expect similar mode of action of the complexes synthesized from this ligand with that of ciprofloxacin. The analysis of the antibacterial data revealed that all the three transition metal complexes exhibited activity ranging from low to high MIZ with 8 ± 0.13 mm at 150 *μ*g/ml for the Ni(II) complex against *E. coli*, and 20.65 ± 0.18 mm at 300 *μ*g/ml for the Cu(II) complex against *P. aeruginosa*. All the three complexes exhibited good activities against *P. aeruginosa* (18.85 ± 0.34, 20.65 ± 0.18, and 15.64 ± 0.22 mm diameter at concentration of 300 *μ*g/ml, respectively) compared to ciprofloxacin with MIZ of 22.98 ± 0.08 mm diameter. These results are in close agreement with previously reported results for related complexes (Indira et al., 2019; Kargar et al., 2021; Sumalatha et al., 2021).

**TABLE 5:** Mean inhibition zone of bacterial growth in mm (mean ± SD).

	Conc. (µg/ml)	Compounds				
		**1**	**2**	**3**	**H**_**3**_ **L**	Ciprofloxacin
*E. coli*	150	10.62 ± 0.36	12.69 ± 0.23	8.00 ± 0.13	6.22 ± 0.14	21.50 ± 0.28
	300	12.00 ± 0.66	13.57 ± 0.29	9.00 ± 0.64	6.50 ± 0.36	22.00 ± 0.50
*P. aeruginosa*	150	16.69 ± 0.18	18.51 ± 0.37	15.07 ± 0.01	6.00 ± 0.25	20.52 ± 0.40
	300	18.85 ± 0.34	20.65 ± 0.18	15.64 ± 0.22	6.24 ± 0.39	22.98 ± 0.08
*S. aureus*	150	11.30 ± 0.17	17.10 ± 0.10	12.50 ± 0.31	0.00 ± 0.00	19.00 ± 0.92
	300	13.22 ± 0.74	17.99 ± 0.03	13.24 ± 0.21	0.00 ± 0.00	20.80 ± 0.37
*S. pyogenes*	150	10.22 ± 0.89	8.50 ± 0.28	0.00 ± 0.00	6.20 ± 0.15	15.90 ± 0.55
	300	12.22 ± 0.66	10.64 ± 0.70	0.00 ± 0.00	7.00 ± 0.11	17.00 ± 0.94

Complexes **1** and **2** have medium to high antibacterial activities with MIZ from 10.62 ± 0.36 to 20.65 ± 0.18 mm at both 150 and 300 *μ*g/ml concentrations of the samples against all the four bacterial strains. However, complex **3** has low to medium MIZ (8 ± 0.13 to 15.64 ± 0.22 mm diameter) at both concentrations against *E. coli*, *P. aeruginosa* and *S. aurous*, but has no antibacterial activity against *S. pyogenes*. Due to the metal’s chelation with the ligand, which encouraged the complexes’ capacity to enter the bacterial strains’ cell membranes, all of the complexes had more antibacterial activity than the precursor ligand (Abd El-Halim et al., 2017; Indira et al., 2019; Sumalatha et al., 2021). From the % activity index data, it can be concluded that Cu(II) complex has higher percent activity indexes (62, 90%) than Zn(II) (54, 82%) and Ni(II) (41, 68%) complexes against both *E. coli* and *P. aeruginosa,* respectively. This is may be due to Cu(II) ion is a borderline Lewis acid and easily bind with similar biochemical such as protein and enzyme by “hard soft acid-base (HSAB)” Principle hence copper complexes showed good binding activity with G. negative (*Pseudomonas aeruginosa)* bacterial strain due to H- bonding interaction with amino acid like arginine having both acid and base end (Table 5). In addition to this, copper (II) coordination compounds can be highly effective in treating microbial infections due to the redox activity of copper ions which interacted with the bacterial chromosome, leading to a decrease in bacterial reproduction (Krasnovskaya et al., 2020).

#### 3.10.2 Antioxidant analysis

**FIGURE 7:**
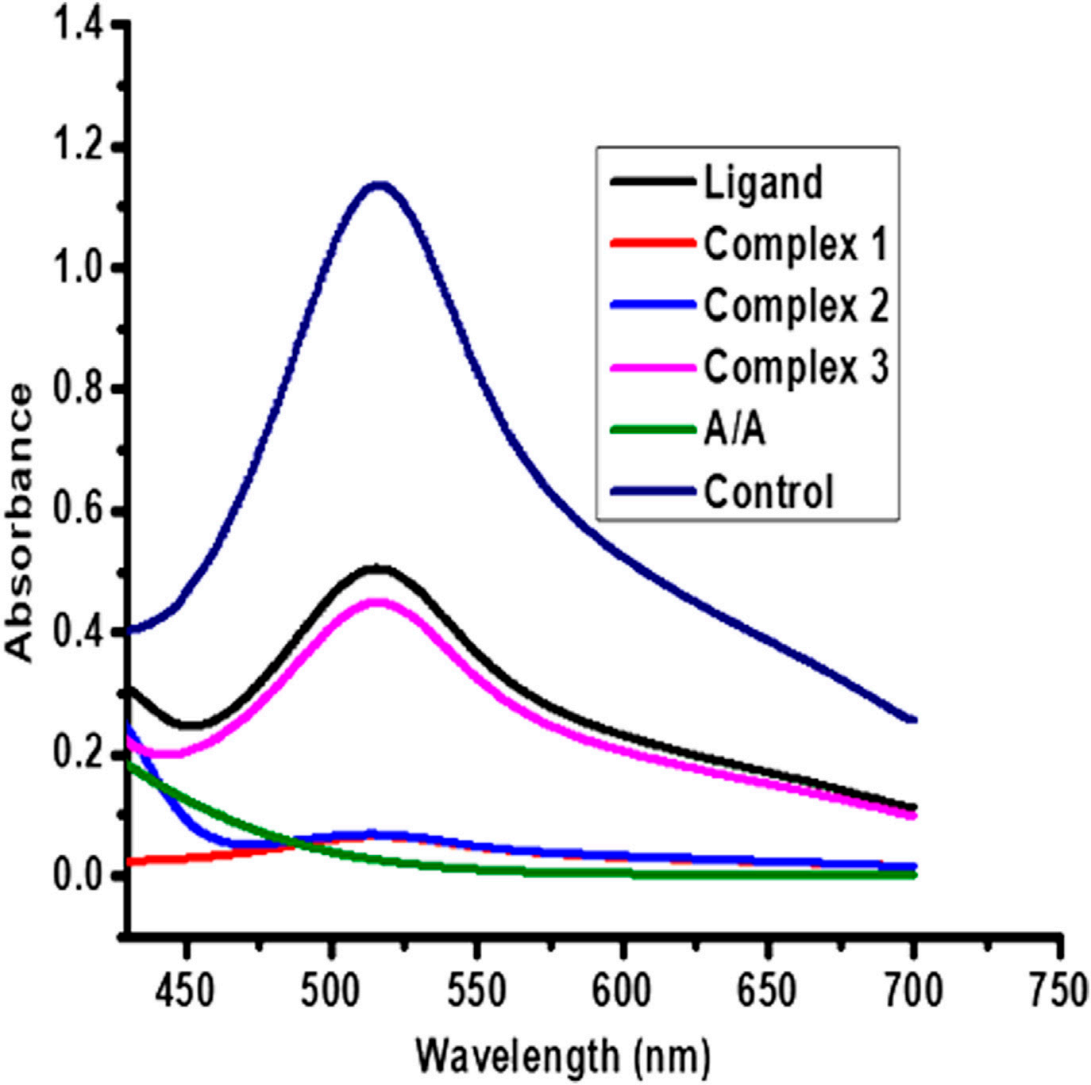
Comparison of absorbance of the control, the reference and the complexes.

The antioxidant activities of the ligand and its complexes **1**–**3** were compared with ascorbic acid as a positive reference (Figure 7). The complexes showed higher antioxidant activities than the corresponding ligand (Supplementary Figure S18A and Supplementary Table S7). This is anticipated to be due to synergetic effects (El-Gammal et al., 2021; Sumalatha et al., 2021), and hence the complexes can potentially be used as radical scavengers. The order follows ascorbic acid > **2** > **1** > **3** > **H**_**3**_ **L**. These results are also in line with the IC_50_ values of 10.46, 8.62, 27.56 and 35.36 μg/ml for complexes **1**–**3** and the ligand, respectively. From the IC_50_ values, complexes **1** and **2** have better antioxidant activities, (Supplementary Figure S18B and Supplementary Table S7). This is may be due to high redox activity, of zinc and copper complex, hence both are important for the formation and functioning of several enzymes and proteins, such as cytochrome C oxidase and Cu/Zn superoxide dismutase, which are involved in the processes of respiration, energy metabolism, and DNA synthesis (Krasnovskaya et al., 2020; Kumar et al., 2020; Damena et al., 2022a), because copper is involved in catalysis (electron transfer), while zinc plays a structural role in these proteins. In addition to, this high activity was probably due to the presence of the OH group in addition to oxidation potential of the metal ions and the decrease of the antioxidant activity of the ligands is indicated that the terminal N-substitution in the ligands does not have any appreciable influence much on the antioxidant properties in agreement with previous reported studies (Ramachandran et al., 2013; Fetoh et al., 2018; Abane-Merzouk et al., 2019; Belkhir-Talbi et al., 2021).

### 3.11 Computational analysis of the compounds

#### 3.11.1 Drug-likeness and ADME predictions

The physicochemical, ADME properties and drug likeness of the ligand and metal complexes are presented in Supplementary Table S8A. The compounds have molecular weights ranging from 259.30 to 400.85 g/mol. The iLogP value of the ligand was found to be 2.22 and that of all the metal complexes showed similar iLogP value of zero compared to ciprofloxacin (2.24). The low value of iLogP for the metal complexes indicates good water solubility of the metal complexes relative to the ligand and the control (Balajee et al., 2016). This is in line with the experimental solubility test results.

The number of hydrogen bond donors of all the compounds range from 2 to 3 (≤5), whereas the number of hydrogen bond acceptors range from 4 to 8 (≤10). The predicted physicochemical properties for drug likeness screening showed that all the synthesized compounds fulfil drug-like molecular nature (Lipinski et al., 2012). Moreover, the topological polar surface area (TPSA) ranges from 56 to 142 Å^2^ for the synthesized compounds. The smaller TPSA value predicted for the Zn(II) complex (57 Å^2^) relative to the ligand’s TPSA value (77.74 Å^2^) is due to the lipophilicity enhancement of the ligand upon coordination with the Zn(II) ion. This phenomenon was also observed in the boiled egg model (Supplementary Figure S13) of the synthesized compounds, in which the compounds follow the lipophilicity order of **1** > **H**_**3**_ **L** > **3** > **2**. It has been reported that compounds with TPSA of 140 Å^2^ and above would be poorly absorbed (<10% fractional absorption) and those with a TPSA 60 Å^2^ would be well absorbed (>90%) (Lipinski et al., 2012; Belkhir-Talbi et al., 2021). From the TPSA data of the synthesized compounds, it is possible to deduce that the ligand and its metal complexes have very good intestinal absorption (Lipinski et al., 2012; Daina et al., 2017)^.^

Skin permeability (logKp) value of the free ligand and its metal complexes were found within the range of −7.10 to −7.53 cms^−1^ (Supplementary Table S8B), deducing that all compounds have low skin permeability (Lipinski et al., 2012; Daina et al., 2017). Similarly, the synthesized compounds were predicted as a substrate of P-glycoprotein (P-gp) which is a transporter and biological barrier and responsible for the ADME of drugs (Daina et al., 2017)^.^ This inferred that the compounds have no tendency to interact with other drugs fingered by the transporter and hence no drug-drug interactions. The inhibition of CYPs leads to toxicity end points (Khojasteh et al., 2011). The high gastrointestinal absorption (GI) together with their fewer tendencies to inhibit cytochrome P450 enzyme family of the liver (CYPs) indicated that the compounds are theoretically non-toxic.

#### 3.11.2 Quantum chemical analysis

The DFT calculated quantum mechanical descriptors are presented in Table 6. The band gap energy (*E*_g_) is correlated with various biological aspects like antibacterial, antioxidant and DNA binding activities (Ali et al., 2019; Ismael et al., 2020). It is also an important stability descriptor (Balajee et al., 2016). A large band gap energy is associated with stable systems, whereas small band gap energy is associated with little stable systems making more reactive compounds (Balajee et al., 2016). The band gap energies of the compounds were found to be 3.834, 4.002, 3.272 and 3.669 eV, respectively for **H**_**3**_ **L**, **1**, **2**, and **3**. The calculated band gap energies for compounds **2** and **3** were found to be less by 0.562 and 0.165 eV, respectively, relative to the ligand (Table 6). A decrease in the band gap energy upon coordination may be associated with the presence of LMCT (Ismael et al., 2020). The band gap energy of complex **1** (4.002 eV) was higher than that of the ligand (3.834 eV) by 0.168 eV, inferring the presence of electron transfer from the HOMO of the metal center to the LUMO of the quinoline part of the ligand (Figure 2).

**TABLE 6:** Quantum chemical descriptors of the ligand and its metal complexes.

Cpds	*E*_HOMO_	*E*_LUMO_	*E*_g_ (eV)	*µ*	*ƞ*	σ	ω	Nu	Dipole moment
H_3_L	−5.960	−2.126	3.834	−4.043	1.917	0.261	4.264	0.235	5.864
1	−6.364	−2.362	4.002	−4.363	2.001	0.250	4.756	0.210	14.413
2	−6.571	−3.299	3.272	−4.935	1.636	0.306	7.445	0.134	19.043
3	−6.303	−2.634	3.669	−4.468	1.834	0.273	5.441	0.184	20.777

Cpds, compounds; E_H_, HOMO energy; E_L_, LUMO energy and Eg = HOMO-LUMO band gap energy.

According to the HSAB principle, soft acids react with soft bases, whereas hard acids react with hard bases (Pearson, 1968; Ismael et al., 2020). Biological molecules such as DNA, proteins, and enzymes are categorized as soft. Hence, the biological activity of a compound increases with increasing softness and decreasing hardness (Pearson, 1968; Ismael et al., 2020). The order of chemical hardness (ɳ) was found to be **1** > **H**_**3**_ **L** > **3** > **2**, suggesting that complex **1** is more stable. This nicely agrees with the TGA analysis (Table 3). Chemical potential (µ) measures the tendency of an electron to escape from equilibrium, and it has been reported that the chemical reactivity of a compound increases with decreasing chemical potential (Ismael et al., 2020; Damena et al., 2022a; Damena et al., 2022b). Chemical potential (µ) is also directly proportional with the Gibbs free energy and related to spontaneity (Table 1). Therefore, the order of chemical reactivity for the synthesized compounds is **2** > **3** > **1** > H_3_ **L** (Table 6), in which compound **2** is higher than that of compounds **3**, **1** and **H**_**3**_ **L** by 0.467, 0.572 and 0.892 eV, respectively. This indicates that compound **2** is more reactive and also nicely agrees with the experimental and molecular docking studies. The dipole moment (in Debye) of the ligand showed large enhancement upon coordination to metal ions (Table 6). This general increase in the dipole moment of the ligand upon coordination is subsequently observed in the antioxidant and antibacterial activity of the synthesized complexes (*vide supra*).

#### 3.11.3 Molecular docking analysis

**FIGURE 8:**
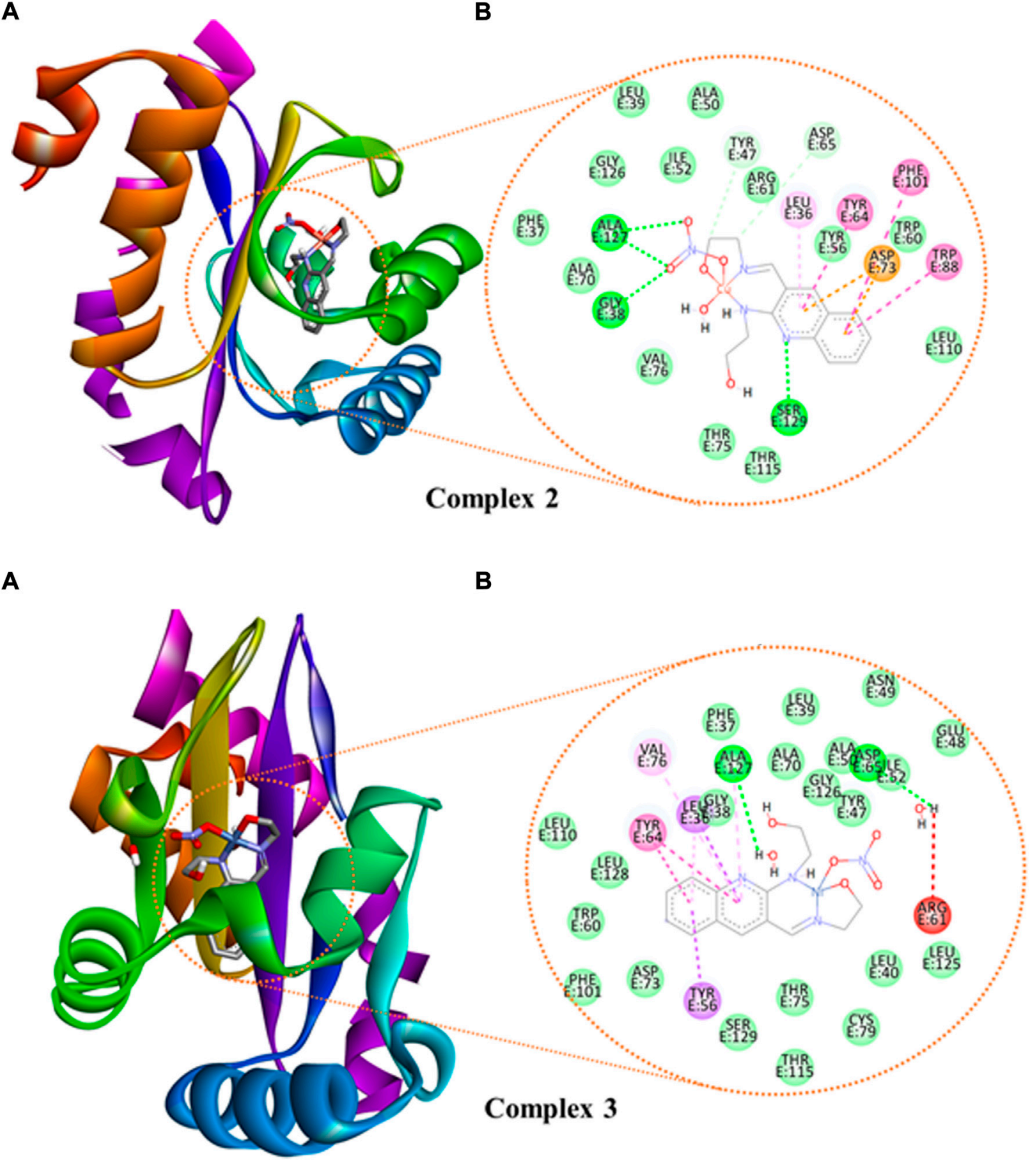
The binding interactions of (**(A)**: 3D and **(B)**: 2D presentations) of complexes **2** and **3** against *P. aeruginosa* LasR.DNA (PDB: 2UV0).

We studied the molecular interaction between the synthesized ligand and its metal complexes against the proteins of *E. coli* DNA gyrase B (PDB ID 6F86) (Supplementary Figure S14) and *P. aeruginosa* LasR (PDB ID: 2UV0) (Supplementary Figures S15, S16 and Figure 8) to understand the mechanism of action. The targeted ligand and its metal complexes interacted with the key amino acids of *E. coli* DNA gyrase B by forming hydrogen bond with Asp-73, Gly-77, Thr-165, and hydrophobic interaction with Ile-78, Ile-94, Glu-50, and Pro-79 within the active sites (Supplementary Figure S14). The results clearly showed that the free hydroxyl chain in the complexes interacted with the amino acids within the active sites of the protein. Among all the reported docking scores, Cu(II) complex showed better docking score, in which the overall *in silico* analysis results revealed the ranking of the complexes as antibacterial agents with the order Cu(II) > Zn(II) > Ni(II) complexes against the *E. coli* DNA gyrase B (Supplementary Table S9). This is also in a good agreement with the experimental *in vitro* antibacterial activity results (Table 5).

We also looked at how the *P. aeruginosa* LasR DNA protein interacted with the synthetic metal complexes to acquire further knowledge. The 2D and 3D representation of the interactions for the compounds and ciprofloxacin are presented in Figure 8 and Supplementary Figures S15, S16, whereas the binding scores and the residual protein-ligand interactions are summarized in Supplementary Table S10. The metal complexes have shown significant interactions within the active sites of the LasR.DNA protein with the key amino acids, for instance Tyr-47, Trp-60, Asp-73, Tyr-64, Leu-36, Trp-88, Arg-61, Thr-75, Cys-79, and Ala-127 (Hussein and Elkhair, 2021). All the investigated compounds showed moderate to equivalent binding scores compared to the clinical drug ciprofloxacin (Supplementary Table S10). The overall *in silico* docking analysis indicated that the Cu(II) complex interacted with the LasR.DNA residues with a binding energy of −8.2 kcal/mol. This result is comparable with the binding energy of ciprofloxacin (−8.00 kcal/mol). Similar docking activity trends were observed for both *E. coli* DNA gyrase B (Supplementary Figure S14) and *P. aeruginosa* LasR (Supplementary Figures S15, S16), all in good agreement with the *in vitro* antibacterial activity results.

## 4 Conclusion

Three new quinoline-based transition metal complexes were synthesized and characterized using PXRD, SEM-EDX, MS, ^1^HNMR, ^13^CNMR, UV-visible spectroscopy, fluorescence spectroscopy, FT-IR, TGA and molar conductance techniques. Density functional theory calculations were used to assist the interpretation of the results. The formation constants of the complexes were found to be in very good agreements with the corresponding thermal stability analysis. All the complexes showed better antibacterial activities than the precursor ligand, particularly the Cu(II) complex showed relatively highest antibacterial activity with mean inhibition zone of 20.65 ± 0.18 mm. Moreover, the Zn(II) and Cu(II) complexes showed better antioxidant activities. The calculated results also showed that none of the target complexed violate Lipinski’s rule of five. The *in silico* drug likeness and molecular docking results agree very well with the corresponding experimental results. The high antibacterial activity of copper complex against Gram-negative bacteria makes the complexes potential alternative drug for treating diseases caused by Gram-negative bacteria after passing cytotoxicity testing. Overall, there is a chance that the ligand and its metal complexes could be further improved for use as medicinal compounds because they have consequently shown promising antioxidant and antibacterial characteristics.

## Data Availability

The original contributions presented in the study are included in the article/Supplementary Material, further inquiries can be directed to the corresponding authors.
