# Supplementary material for: Synthesis, characterization, and biological activities of zinc(II), copper(II) and nickel(II) complexes of an aminoquinoline derivative
Source: Front Chem. 2022 Nov 3;10:1053532. doi: 10.3389/fchem.2022.1053532 (PMC9669718; doi:10.3389/fchem.2022.1053532)
Supplement: Supplementary file 1 [file DataSheet1.PDF]

SUPPLEMENTARY INFORMATION

Synthesis, Characterization, and Biological Activities of Zinc(II), Copper(II) and Nickel(II) Complexes of an Aminoquinoline Derivative

Tadewos Damena[1,2]*, Mamaru Bitew Alem[1], Digafie Zeleke[3], Tegene Desalegn[1]*, Rajalakshmanan Eswaramoorthy[4] and Taye B. Demissie[5]*

[1] Department of Applied Chemistry, Adama Science and Technology University, P.O.Box 1888, Adama, Ethiopia.

[2]Department of Chemistry, Wachemo University, P.O.Box 667, Hossana, Ethiopia

[3] Department of Chemistry, Salale University, P.O.Box 245, Fitche, Ethiopia.

[4] Department of Biomaterials, Saveetha University, Chennai-600 077, India.

[5] Department of Chemistry, University of Botswana, Notwane Rd, P/bag UB 00704 Gaborone, Botswana

Correspondence: btadeows@gmail.com (Damena),

ORCID Id: https://orcid.org/**:0000-0001-5345-002X**

tegened@yahoo.com (Desalegn),

ORCID Id: https://orcid.org/**:0000-0003-0239-8326**

sene3095@gmail.com (Demissie),

ORCID Id: https://orcid.org/**:0000-0001-8735-4933**

List of Figures

Figure S1. ^{1}HNMR spectrum of 2-(((2-((2-hydroxyethyl)amino)quinolin-3-yl)methylene)amino)ethan-1-ol 3
Figure S2: ^{13}CNMR spectrum of 2-(((2-((2-hydroxyethyl)amino)quinolin-3-yl)methylene)amino)ethan-1-ol. 4
Figure S3. DEPT-135 spectrum of 2-(((2-((2-hydroxyethyl)amino)quinolin-3-yl)methylene)amino)ethan-1-ol 5
Figure S4. EDX spectrum of: A, Ni(II) complex, B, Ligand and SEM- image of: C, Ni(II) complex, D, Ligand........ 6
Figure S5. Procedure and image of synthesized complexes 6
Figure S6. Job's curves at 25, 30, 37 and 40 ^{o}C for complexes **1- 3** 7
Figure S7. Plot of lnK versus 1/T for complexes........ 7
Figure S8. FT-IR spectra of: A) ligand and B) Zn(II) metal complexes. 8
Figure S9. FT-IR spectra of: C) Cu(II) and D) Ni(II), metal complexes 9
Figure S10. Fluorescence spectra of: ligand, a) Zn(II), b) Cu(II), and c) Ni(II) complexes... 10
Figure S11. Mass spectra of: A) Zn(II), B) Cu(II) and C) Ni(II) complexes........ 11
Figure S12. TGA curves of: A) Zn(II) B) Cu(II) and C) Ni(II) complexes 12
Figure S13. Boiled egg model representation of studied compounds........ 13
Figure S14. The binding interactions of the compounds against *E.coli* DNA gyrase (PDB ID: 6F86). 14
Figure S15. The binding interactions of: compounds against *P. aeruginosa* LasR.DNA binding domain (PDB: 2UV0). 15
Figure S16. The binding interactions of: compounds against *P. aeruginosa* LasR.DNA binding domain (PDB: 15
Figure S17. The optimized structure of the complexes 16
Figure S18 Radical scavenging activities (A), and IC_{50} of the reference and the complexes (B) 17

List of Tables

Table S1: One-way analysis of variance 18
Table S2 Physicochemical properties of the ligand, Zn(II), Cu(II) and Ni(II) compounds 19
Table S3: Metal–ligand formation constants and stoichiometric 19
Table S4: FTIR data of the ligand and its Zn(II), Cu(II) and Ni(II) complexes. The B3LYP-GD3/6-311++G**/LanL2DZ calculated results are presented in parenthesis.[a] 20
Table S5: Estimated lattice parameters (a, b, c, α, β and γ) of the complexes **1** –**3**. 21
Table S6: Powder XRD data and estimated Miller indices (hkl) of the complexes **1**–**3**........ 21
Table S7: Radical scavenging activity of the synthesised compounds (mean ± SD) 22
Table S8: ADME and Drug likeness descriptors of Ligand and its complexes. 22
Table S9: Molecular docking of ligand and complexes against *E. Coli* DNA Gyrase (PDB ID 6f86). 23
Table S10: Molecular docking scores and residual amino acid interactions of metal complexes against *P. aeruginosa* LasR.DNA binding domain (PDB: 2UV0). 23
Table S11: B3LYP-GD3/311++G(d,p)/LanL2DZ/PCM/methanol optimized geometries..... 24

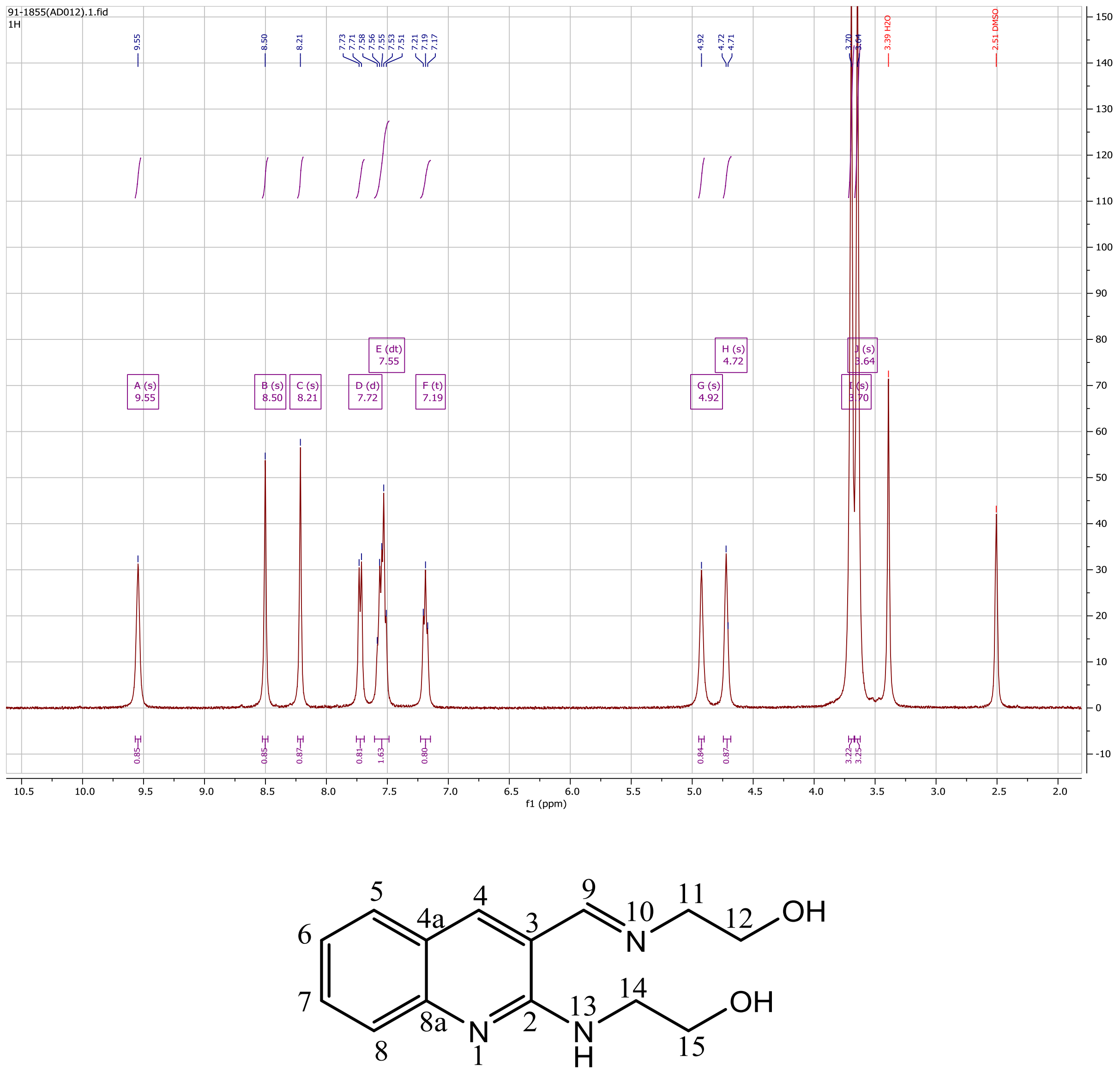

Figure S1. ^{1}HNMR spectrum of 2-(((2-((2-hydroxyethyl)amino)quinolin-3-yl)methylene)amino)ethan-1-ol

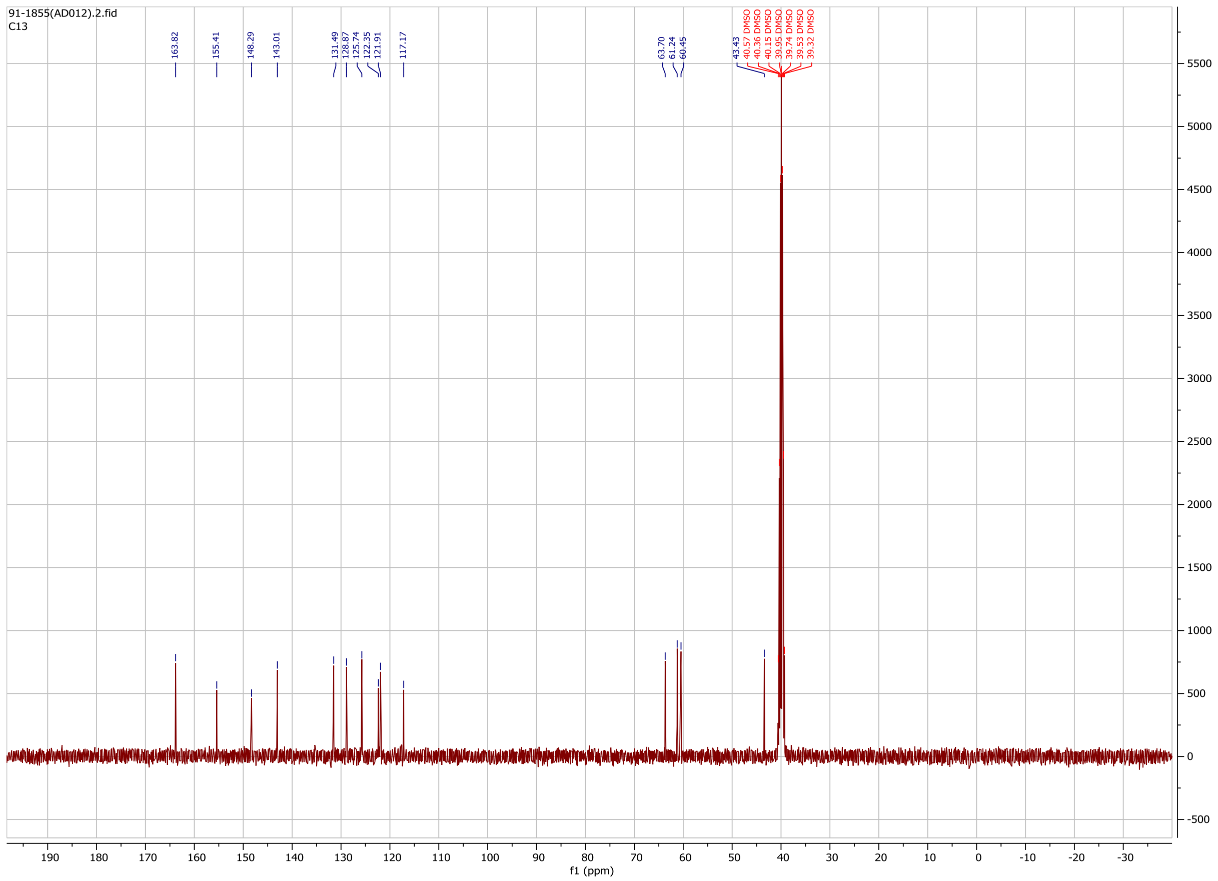

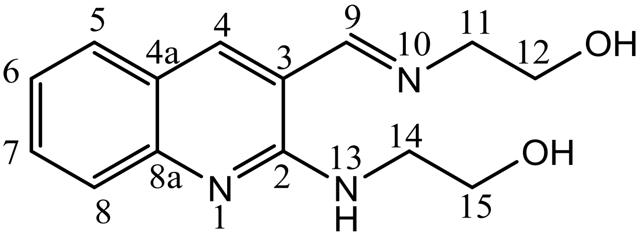

Figure S2: ^{13}CNMR spectrum of 2-(((2-((2-hydroxyethyl)amino)quinolin-3-yl)methylene)amino)ethan-1-ol.

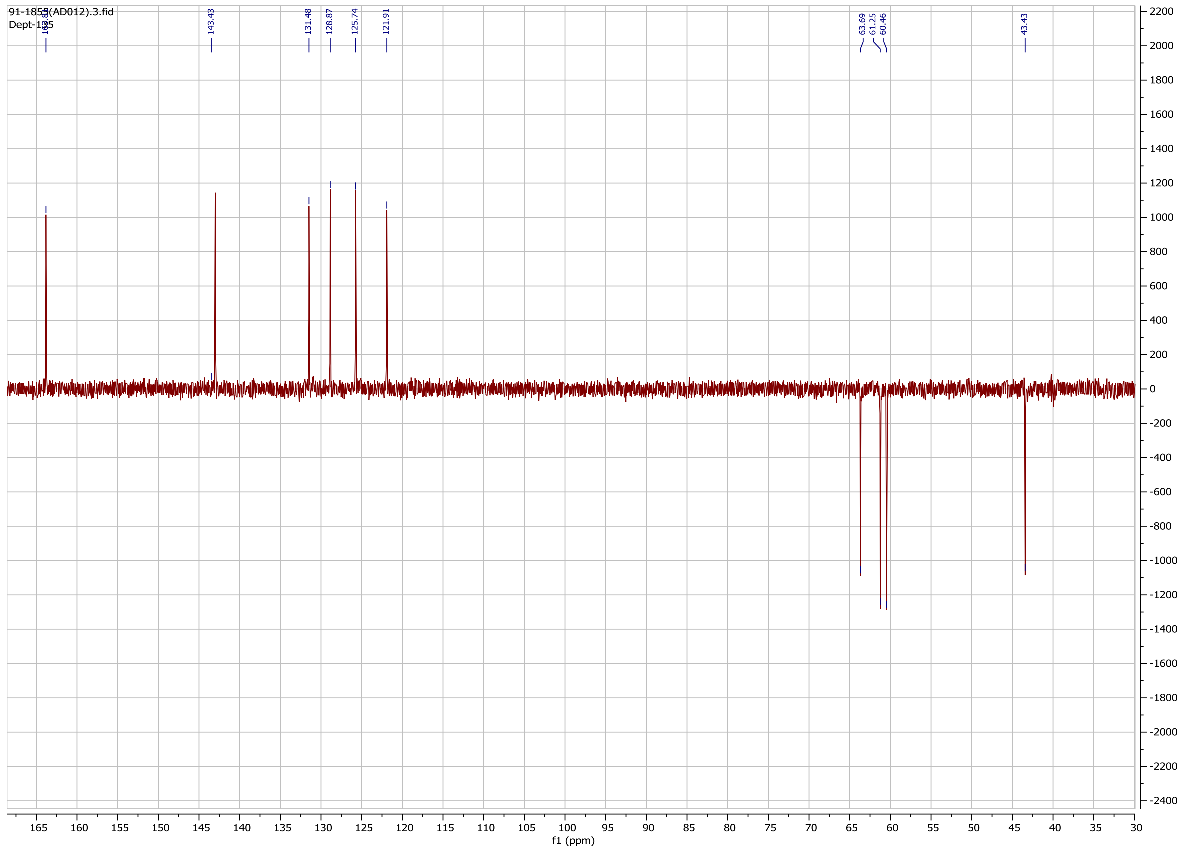

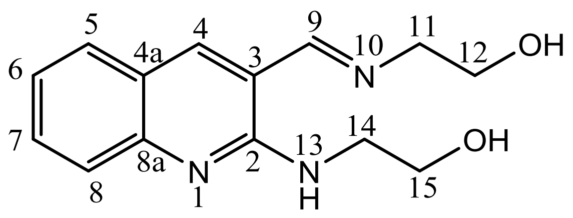

Figure S3. DEPT-135 spectrum of 2-(((2-((2-hydroxyethyl)amino)quinolin-3-yl)methylene)amino)ethan-1-ol

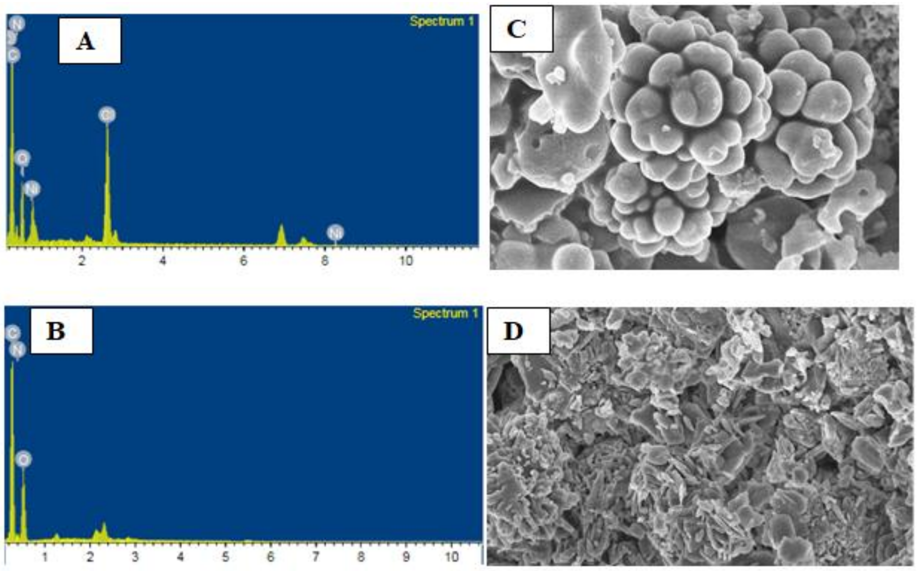

Figure S4. EDX spectrum of: A, Ni(II) complex, B, Ligand and SEM- image of: C, Ni(II) complex, D, Ligand.

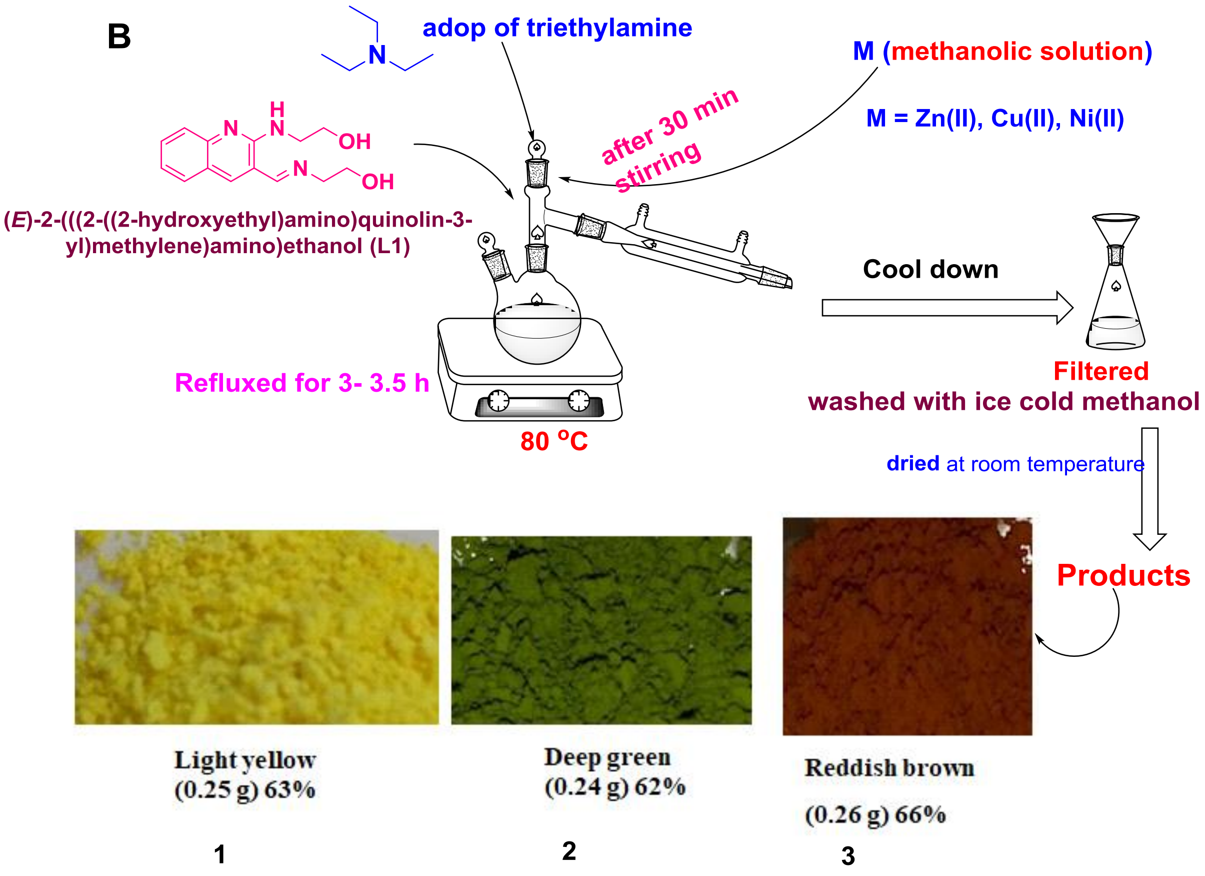

Figure S5. Procedure and image of synthesized complexes

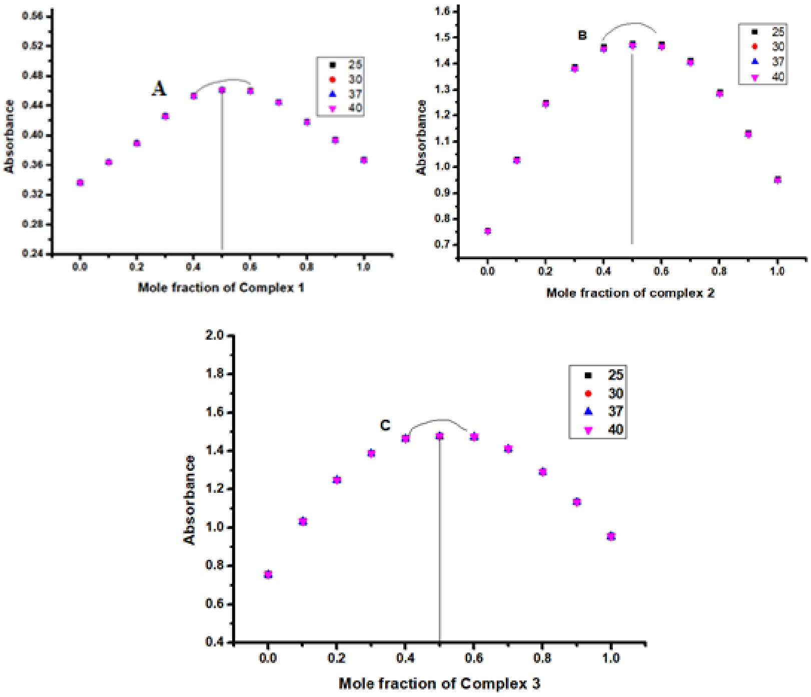

Figure S6. Job's curves at 25, 30, 37 and 40 ^{o}C for complexes **1- 3**

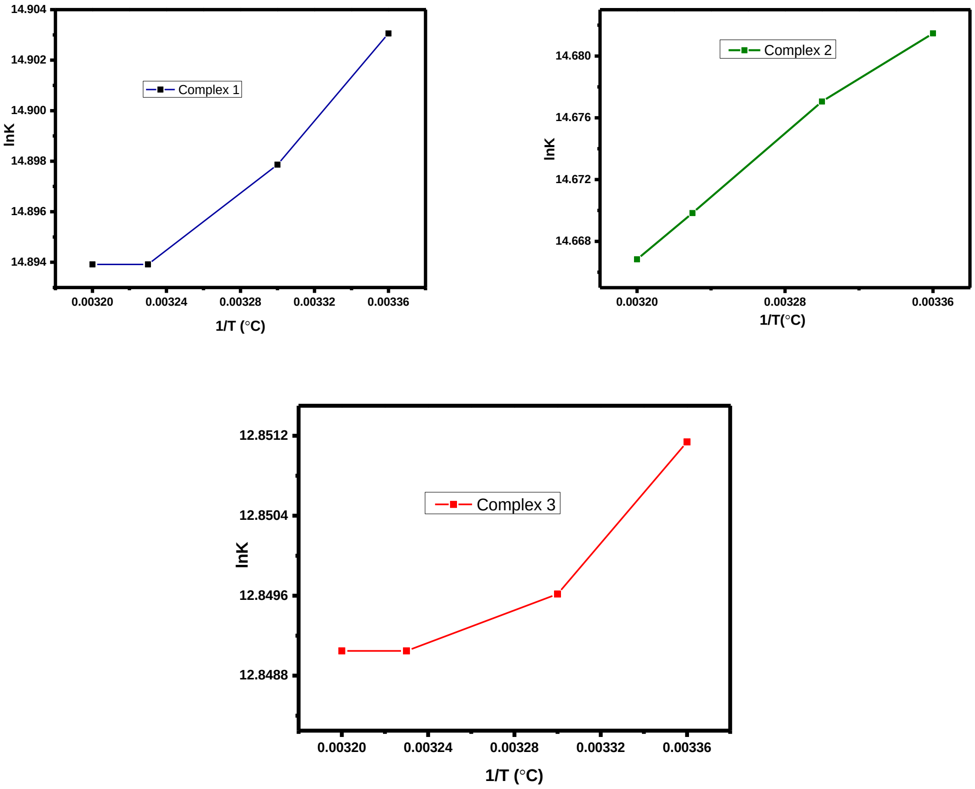

Figure S7. Plot of lnK versus 1/T for complexes.

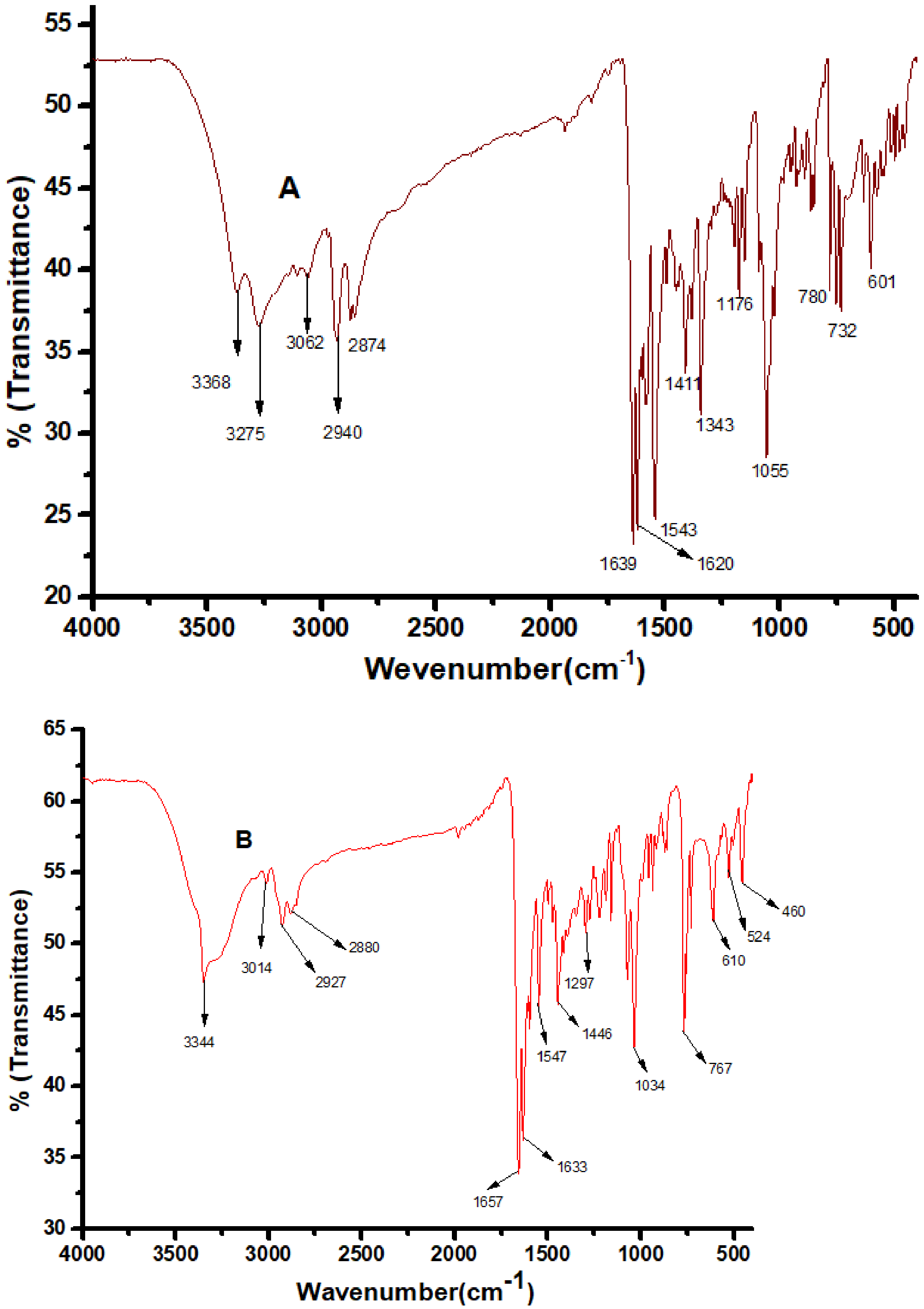

Figure S8. FT-IR spectra of: A) ligand and B) Zn(II) metal complexes.

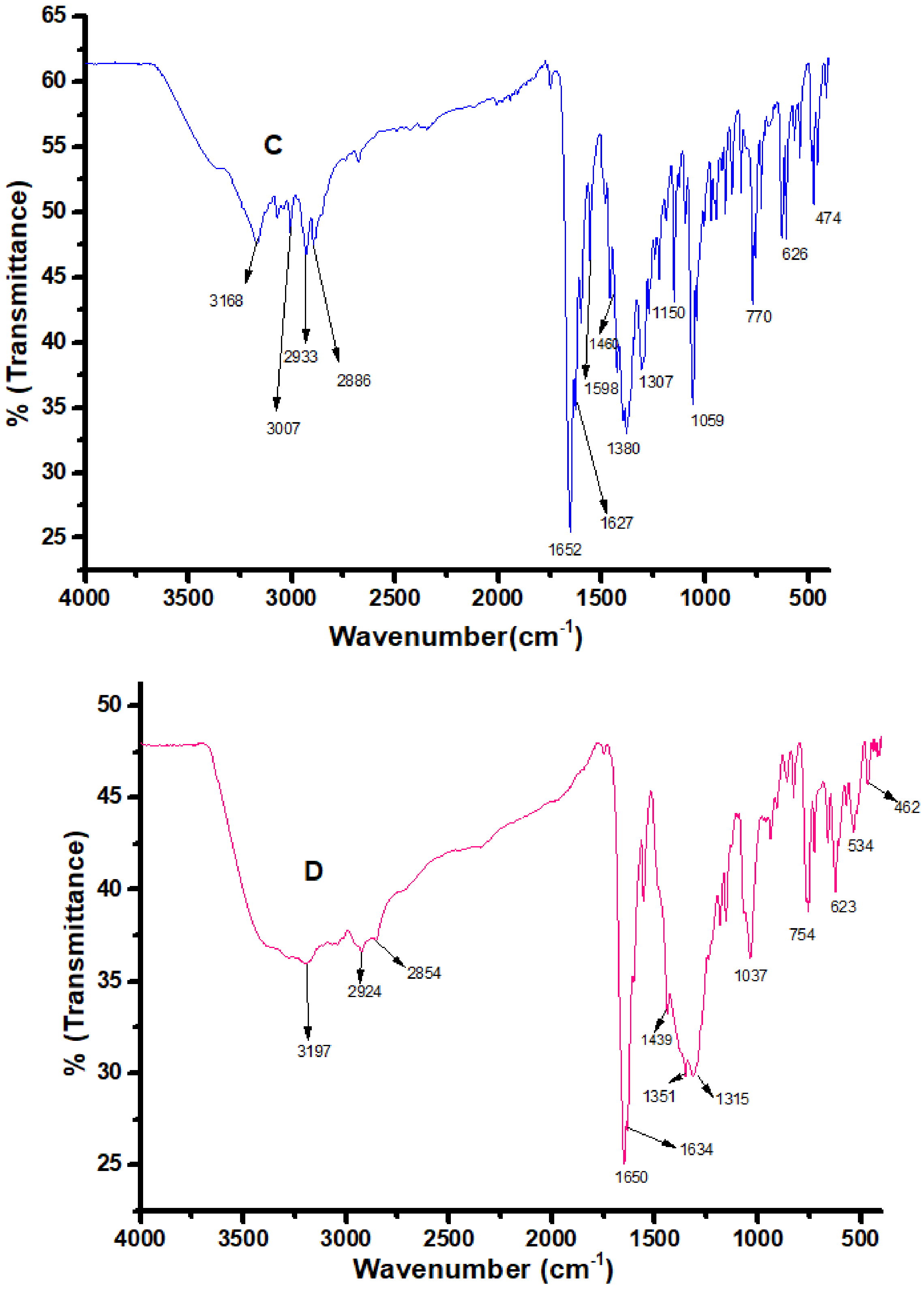

Figure S9. FT-IR spectra of: C) Cu(II) and D) Ni(II), metal complexes

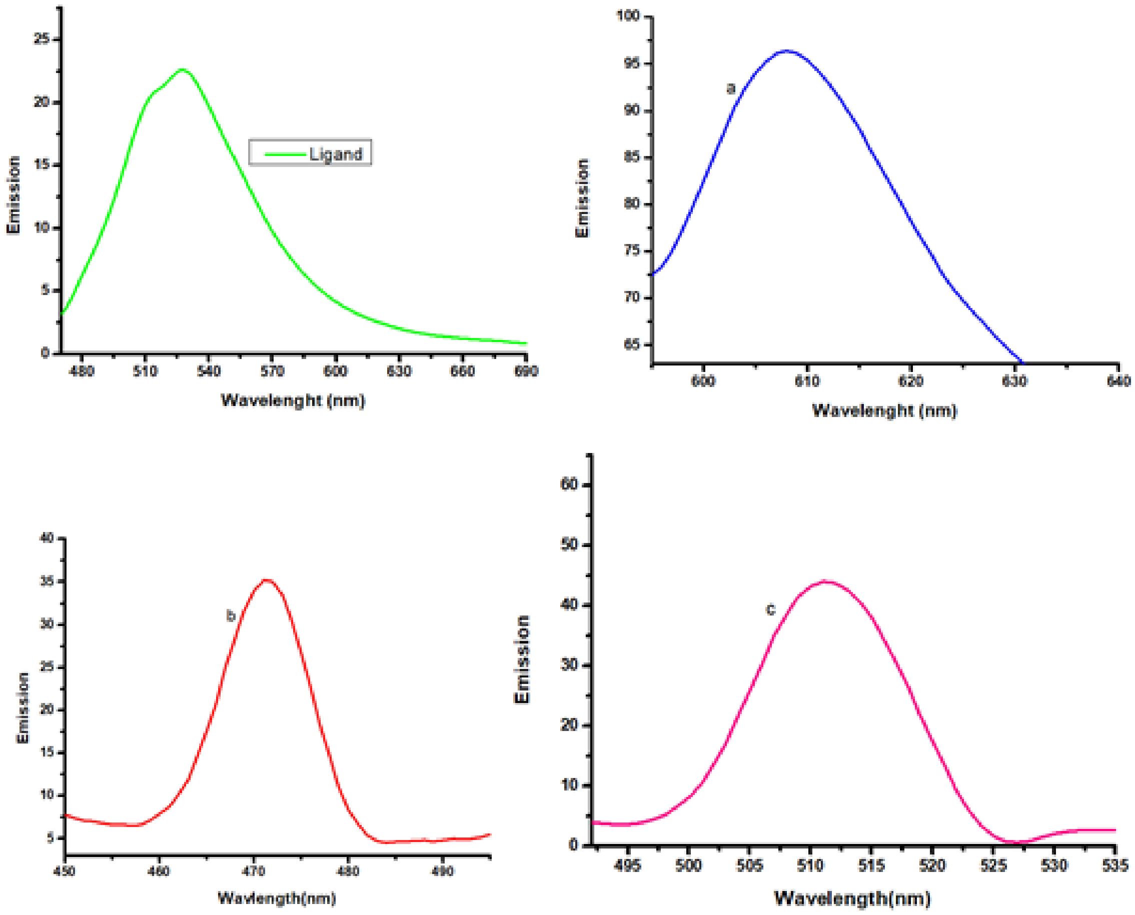

Figure S10. Fluorescence spectra of: ligand, a) Zn(II), b) Cu(II), and c) Ni(II) complexes

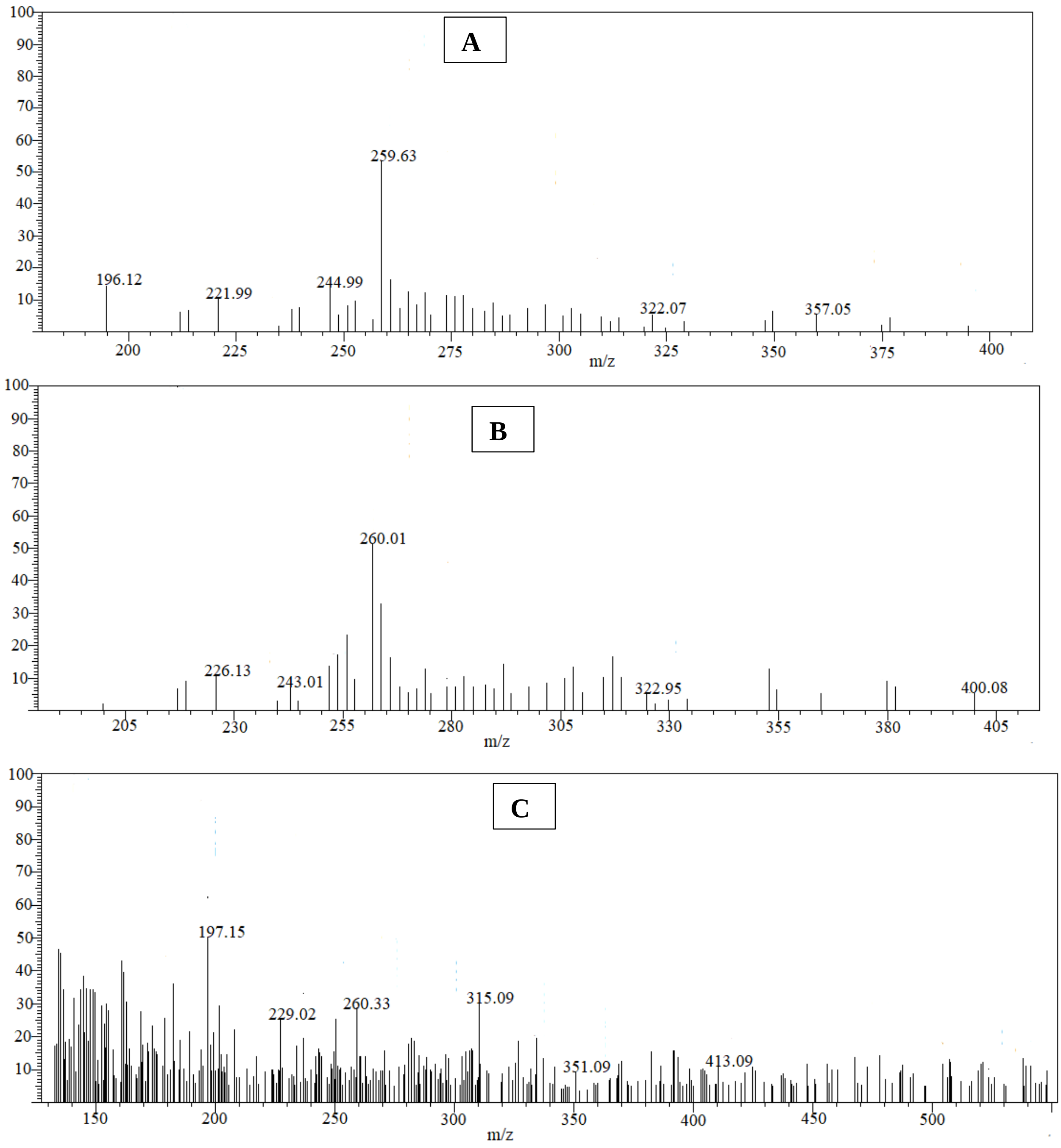

Figure S11. Mass spectra of: A) Zn(II), B) Cu(II) and C) Ni(II) complexes.

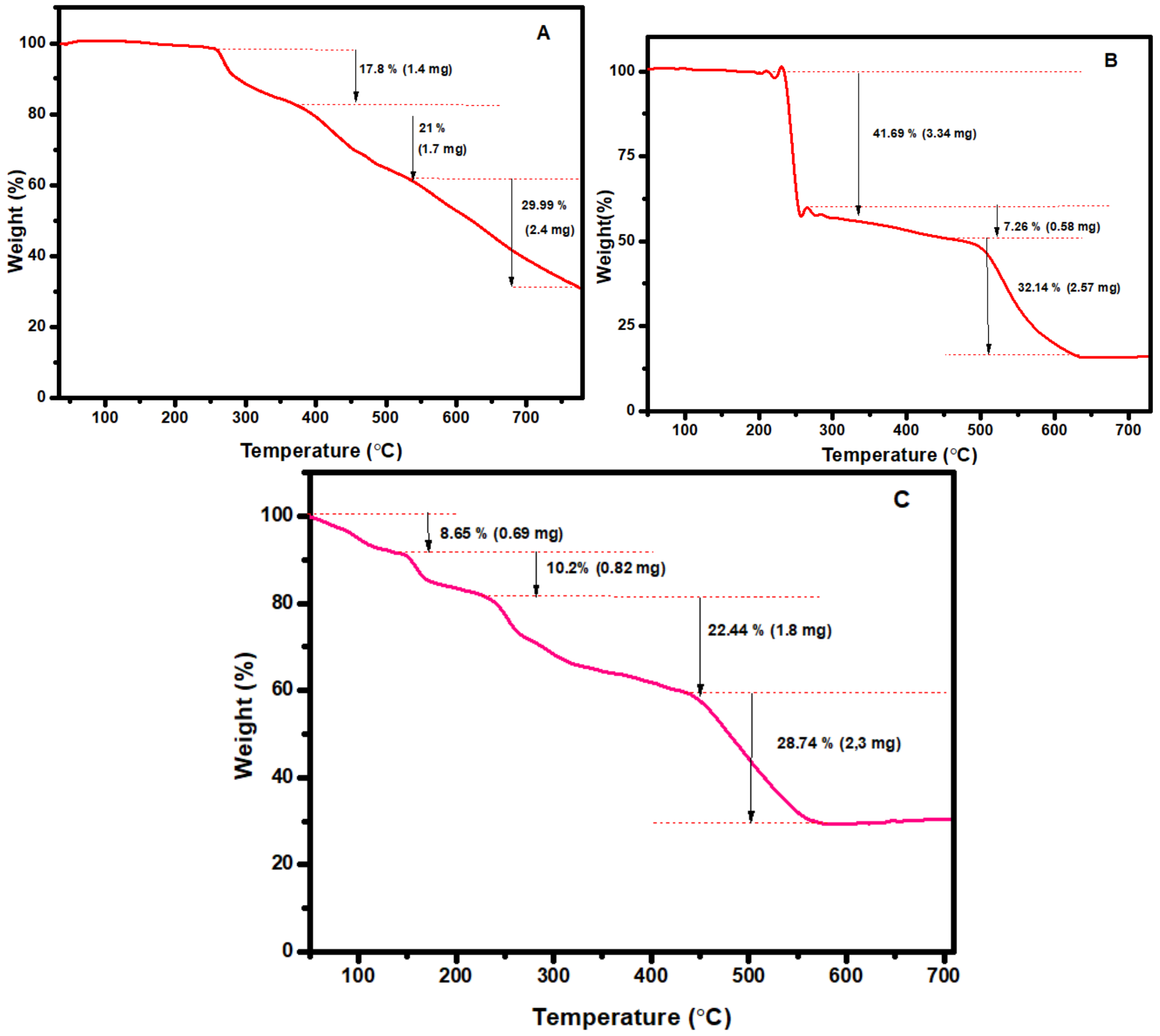

Figure S12. TGA curves of: A) Zn(II) B) Cu(II) and C) Ni(II) complexes

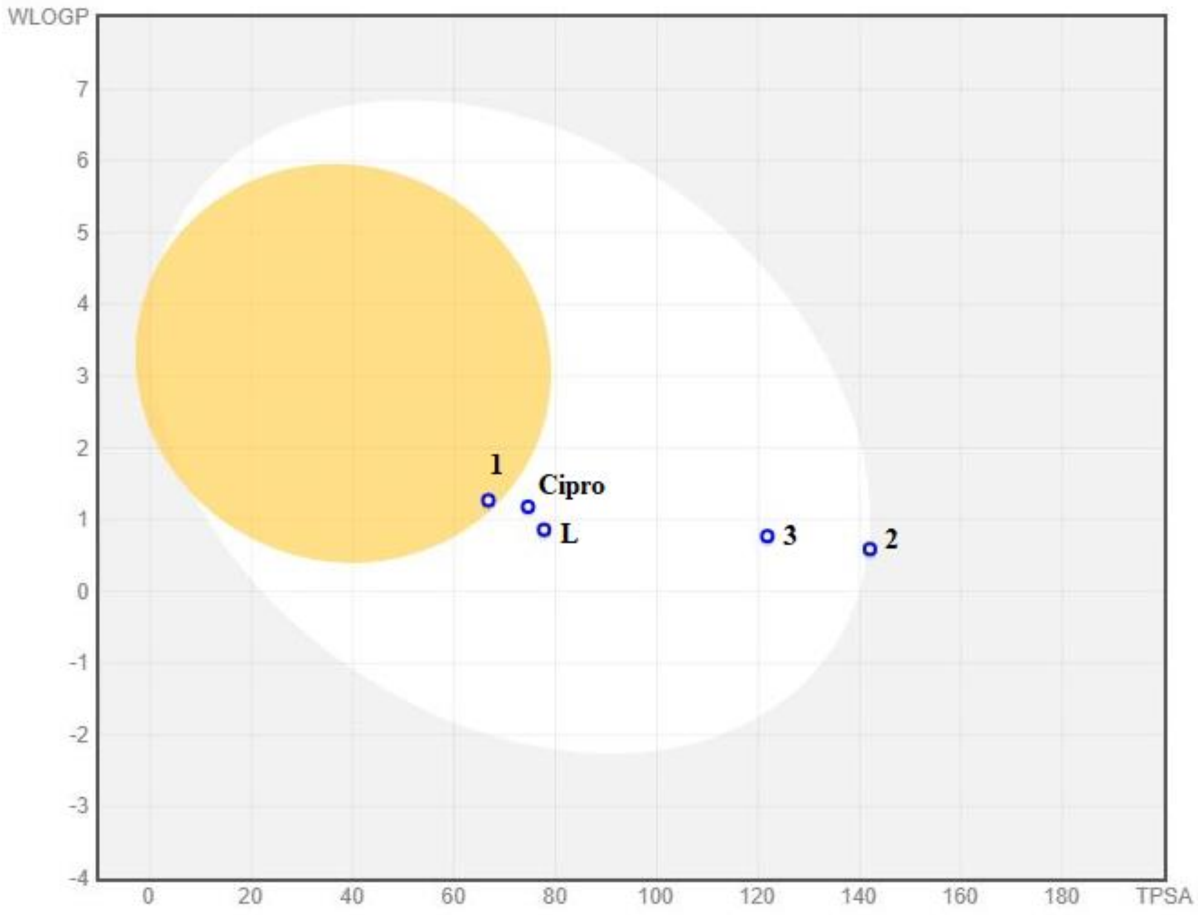

Figure S13. Boiled egg model representation of studied compounds.

Note that Cipro stands for the positive control ciprofloxacin

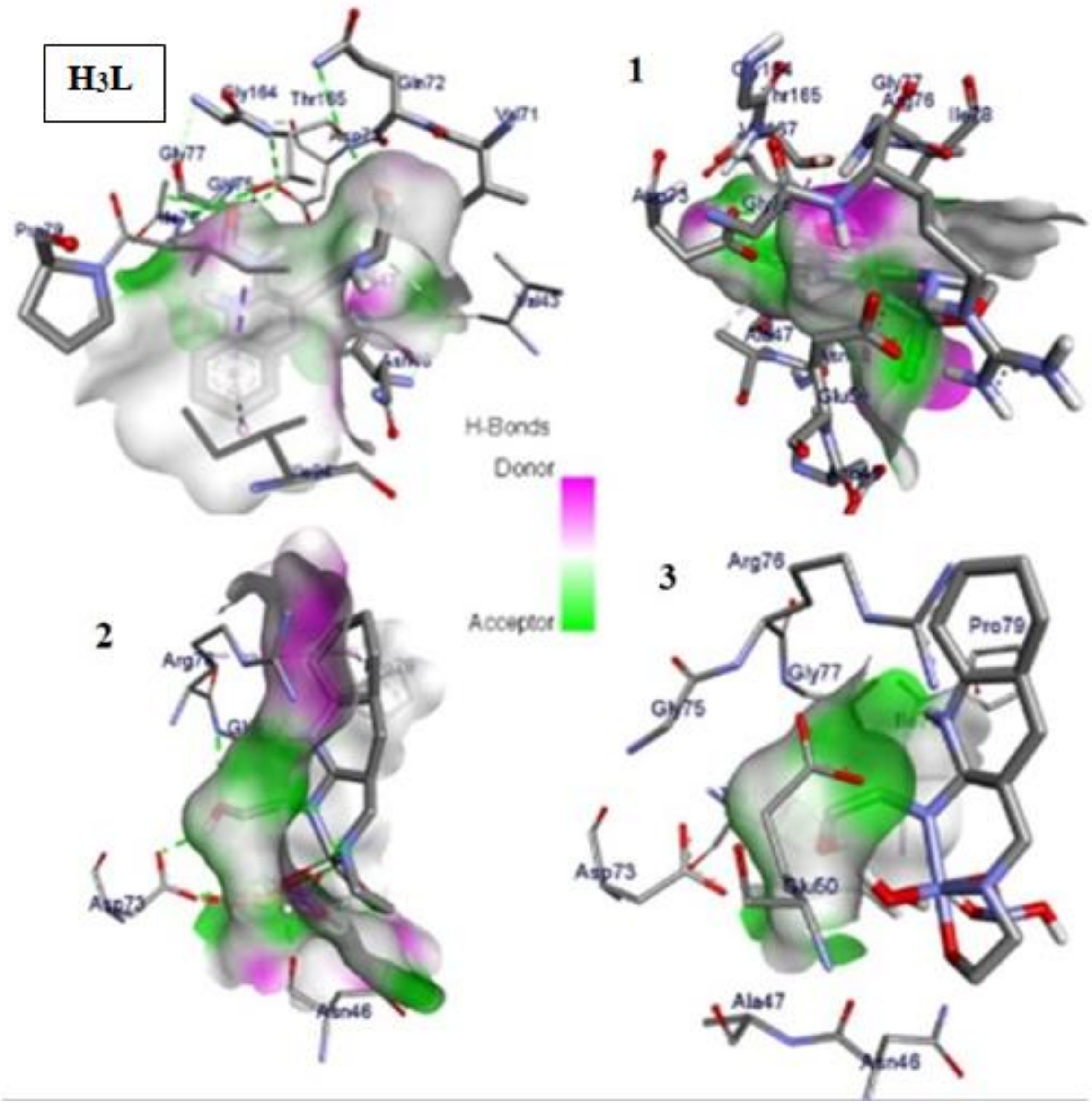

Figure S14. The binding interactions of the compounds against *E.coli* DNA gyrase (PDB ID: 6F86).

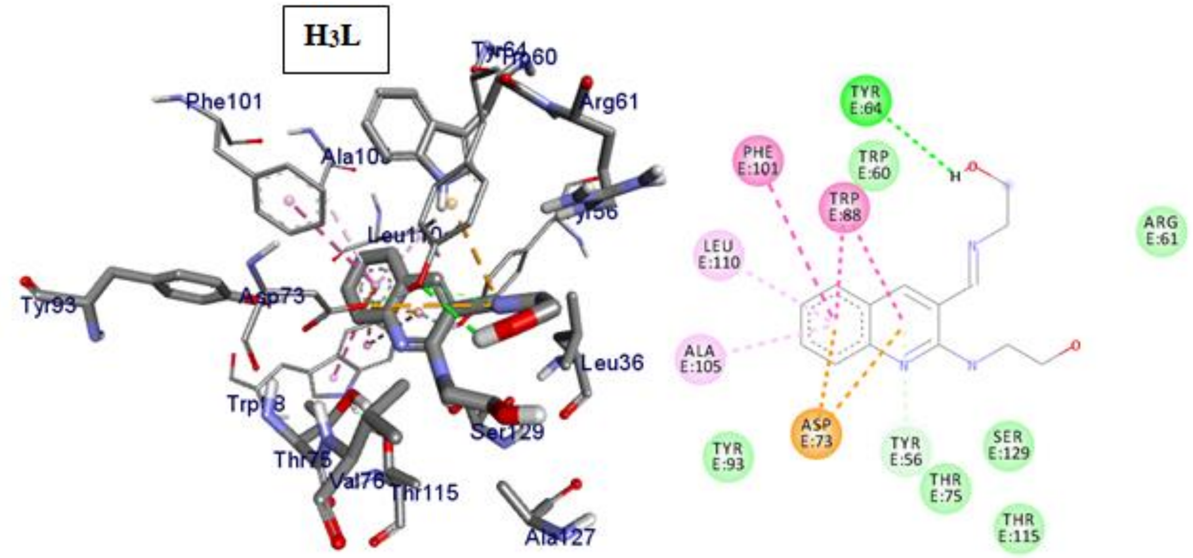

Figure S15. The binding interactions of: compounds against *P. aeruginosa* LasR.DNA binding domain (PDB: 2UV0).

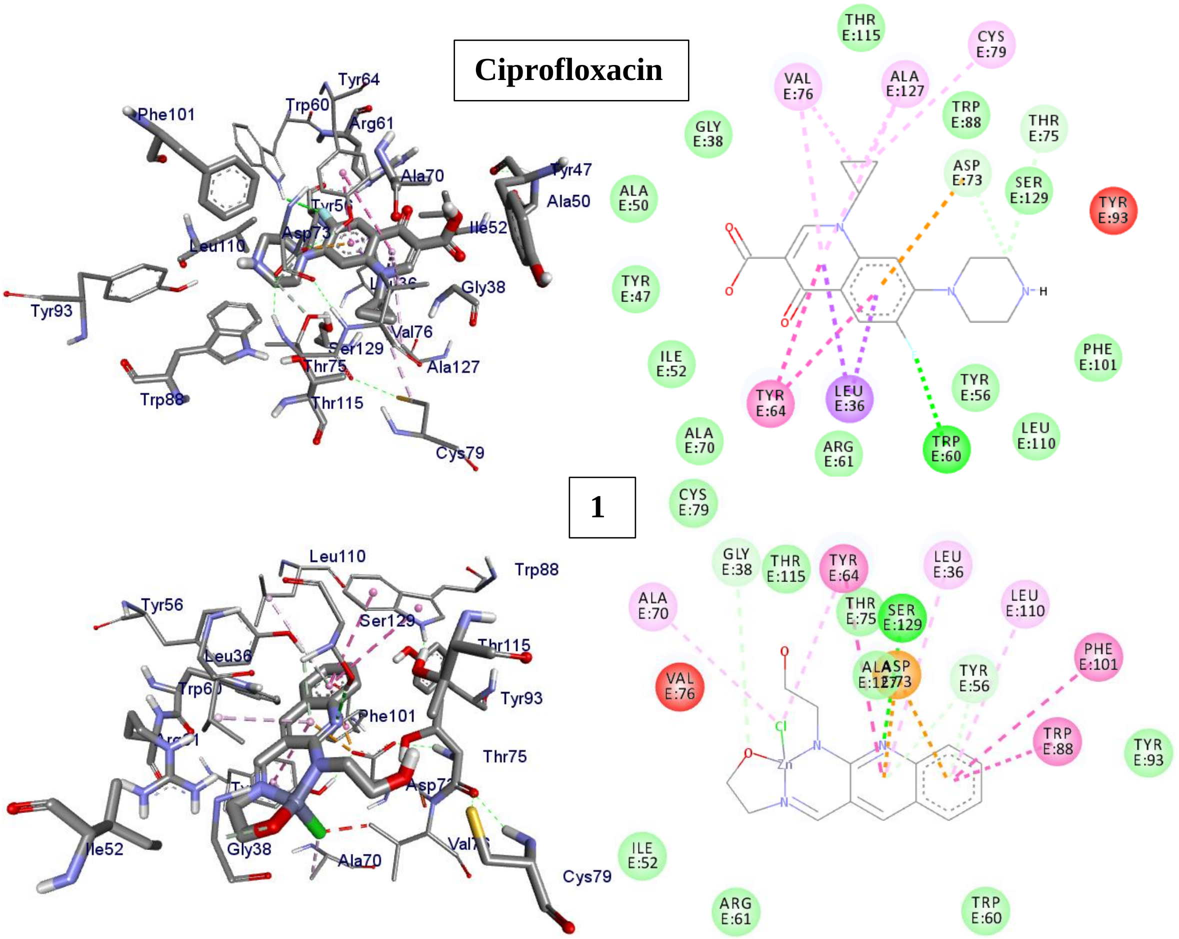

Figure S16. The binding interactions of: compounds against *P. aeruginosa* LasR.DNA binding domain (PDB:

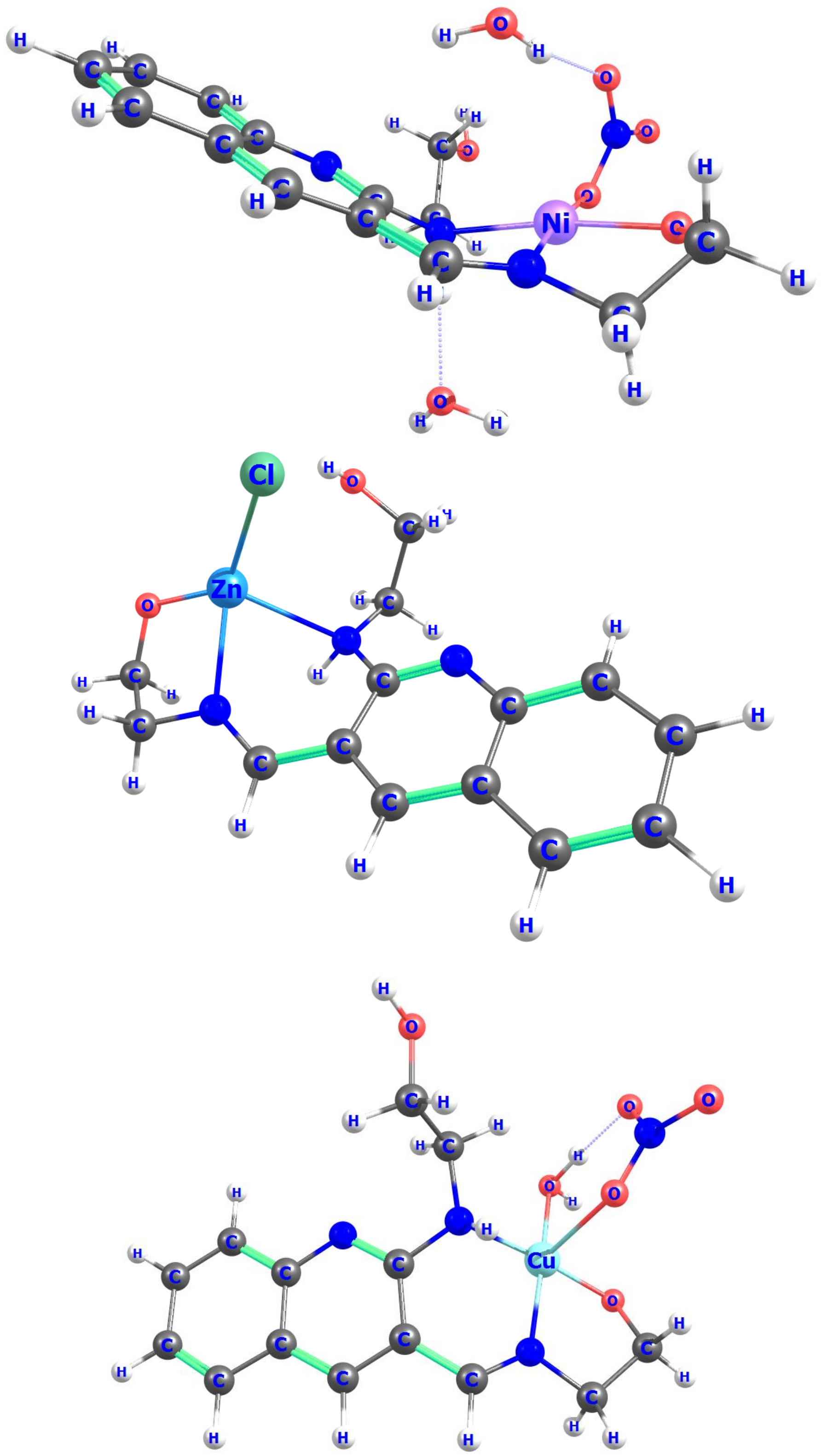

Figure S17. The optimized structure of the complexes

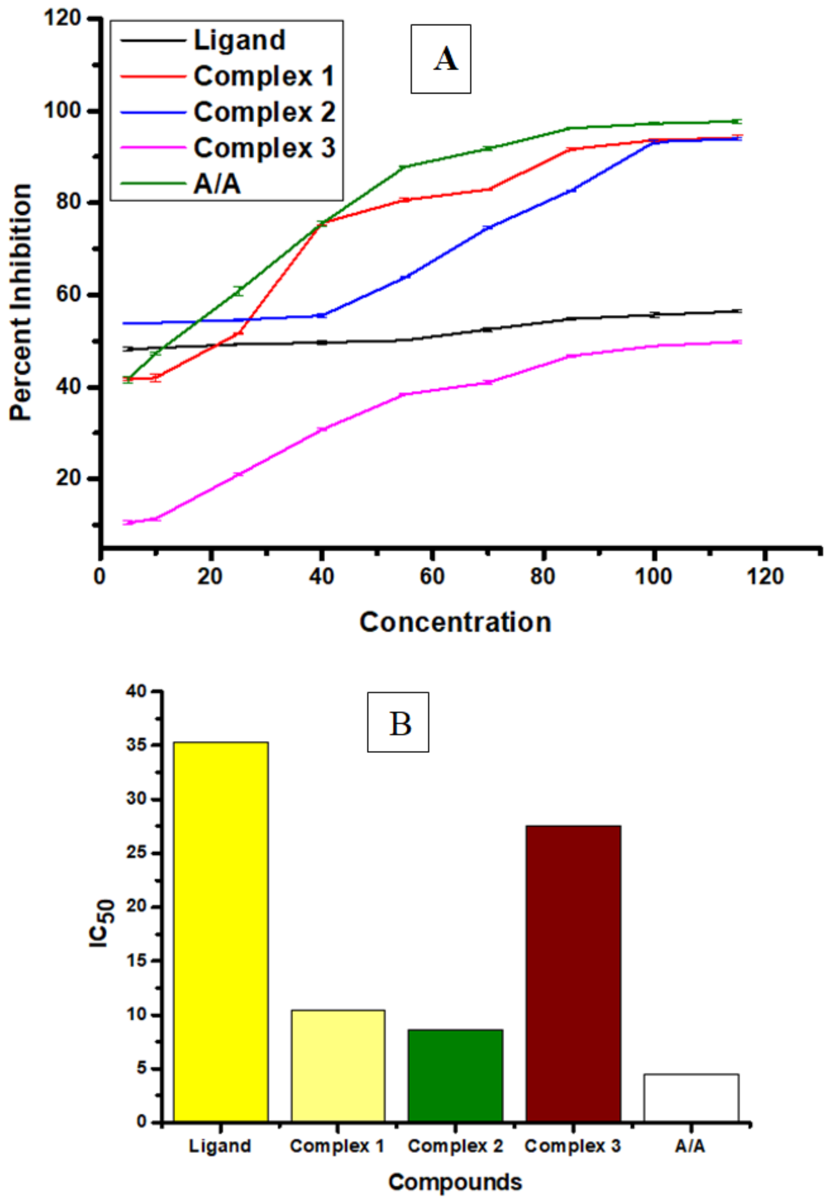

Figure S18 Radical scavenging activities (A), and IC_{50} of the reference and the complexes (B)

Table S1: One-way analysis of variance

Table Analyzed	E. Coli				
One-way analysis of variance					
P value	< 0.0001				
P value summary	***				
Are means signif. different? (P < 0.05)	Yes				
Number of groups	7				
F	476.6				
R squared	0.9951				
ANOVA Table	SS	df	MS		
Treatment (between columns)	476.1	6	79.34		
Residual (within columns)	2.331	14	0.1665		
Total	478.4	20			
Bonferroni's Multiple Comparison Test	Mean Diff.	t	Significant? P < 0.05?	Summary	95% CI of diff
1 vs 2	-2.070	6.213	Yes	***	-3.302 to -0.8376
1 vs 3	2.620	7.864	Yes	***	1.388 to 3.852
1 vs Ligand	4.400	13.21	Yes	***	3.168 to 5.632
1 vs Ciprofloxacin	-10.88	32.66	Yes	***	-12.11 to -9.648
2 vs 3	4.690	14.08	Yes	***	3.458 to 5.922
2 vs Ligand	6.470	19.42	Yes	***	5.238 to 7.702
2 vs Ciprofloxacin	-8.810	26.44	Yes	***	-10.04 to -7.578
3 vs Ligand	1.780	5.343	Yes	**	0.5476 to 3.012
3 vs Ciprofloxacin	-13.50	40.52	Yes	***	-14.73 to -12.27
Ligand vs Ciprofloxacin	-15.28	45.86	Yes	***	-16.51 to -14.05
Table Analyzed	P. Aeruginosa				
One-way analysis of variance					
P value	< 0.0001				
P value summary	***				
Are means signif. different? (P < 0.05)	Yes				
Number of groups	7				
F	3040				
R squared	0.9992				
ANOVA Table	SS	df	MS		
Treatment (between columns)	1034	6	172.3		
Residual (within columns)	0.7936	14	0.05669		
Total	1035	20			
Tukey's Multiple Comparison Test	Mean Diff.	q	Significant? P < 0.05?	Summary	95% CI of diff
1 vs 2	-1.820	13.24	Yes	***	-2.484 to -1.156
1 vs 3	1.620	11.79	Yes	***	0.9562 to 2.284
1 vs Ligand	10.69	77.77	Yes	***	10.03 to 11.35
1 vs Ciprofloxacin	-3.830	27.86	Yes	***	-4.494 to -3.166
2 vs 3	3.440	25.03	Yes	***	2.776 to 4.104
2 vs Ligand	12.51	91.01	Yes	***	11.85 to 13.17
2 vs Ciprofloxacin	-2.010	14.62	Yes	***	-2.674 to -1.346
3 vs Ligand	9.070	65.98	Yes	***	8.406 to 9.734
3 vs Ciprofloxacin	-5.450	39.65	Yes	***	-6.114 to -4.786
Ligand vs Ciprofloxacin	-14.52	105.6	Yes	***	-15.18 to -13.86
Table Analyzed	S. Aureus				
One-way analysis of variance					
P value	< 0.0001				
P value summary	***				
Are means signif. different? (P < 0.05)	Yes				
Number of groups	7				
F	767.5				
R squared	0.9970				
ANOVA Table	SS	df	MS		
Treatment (between columns)	723.6	6	120.6		
Residual (within columns)	2.200	14	0.1571		
Total	725.8	20			
Tukey's Multiple Comparison Test	Mean Diff.	q	Significant? P < 0.05?	Summary	95% CI of diff
1 vs 2	-5.800	25.34	Yes	***	-6.905 to -4.695
1 vs 3	-1.200	5.243	Yes	*	-2.305 to -0.09479
1 vs Ligand	11.30	49.37	Yes	***	10.19 to 12.41
1 vs Ciprofloxacin	-7.700	33.64	Yes	***	-8.805 to -6.595
2 vs 3	4.600	20.10	Yes	***	3.495 to 5.705
2 vs Ligand	17.10	74.72	Yes	***	15.99 to 18.21
2 vs Ciprofloxacin	-1.900	8.302	Yes	***	-3.005 to -0.7948
3 vs Ligand	12.50	54.62	Yes	***	11.39 to 13.61
3 vs Ciprofloxacin	-6.500	28.40	Yes	***	-7.605 to -5.395
Ligand vs Ciprofloxacin	-19.00	83.02	Yes	***	-20.11 to -17.89
Table Analyzed	S. Pyogenes				
One-way analysis of variance					
P value	< 0.0001				
P value summary	***				
Are means signif. different? (P < 0.05)	Yes				
Number of groups	7				
F	673.1				
R squared	0.9965				

ANOVA Table	SS	df	MS		
Treatment (between columns)	689.7	6	115.0		
Residual (within columns)	2.391	14	0.1708		
Total	692.1	20			
Tukey's Multiple Comparison Test	Mean Diff.	q	Significant? P < 0.05?	Summary	95% CI of diff
1 vs 2	1.720	7.209	Yes	**	0.5678 to 2.872
1 vs 3	10.22	42.83	Yes	***	9.068 to 11.37
1 vs Ligand	4.020	16.85	Yes	***	2.868 to 5.172
1 vs Ciprofloxacin	-5.680	23.81	Yes	***	-6.832 to -4.528
2 vs 3	8.500	35.62	Yes	***	7.348 to 9.652
2 vs Ligand	2.300	9.640	Yes	***	1.148 to 3.452
2 vs Ciprofloxacin	-7.400	31.01	Yes	***	-8.552 to -6.248
3 vs Ligand	-6.200	25.99	Yes	***	-7.352 to -5.048
3 vs Ciprofloxacin	-15.90	66.64	Yes	***	-17.05 to -14.75
Ligand vs Ciprofloxacin	-9.700	40.65	Yes	***	-10.85 to -8.548

Table S2 Physicochemical properties of the ligand, Zn(II), Cu(II) and Ni(II) compounds

	Color	Yield (%)	Melting point (^oC)	Molar conductance ($\Omega^{-1}mol^{-1}cm^2$ 25 ^oC)
$C_{14}H_{17}N_3O_2$ (L)	Yellow	(2.91 g) 86	80 – 85	0
$[Zn(H_2L)Cl]$ (1)	Light yellow	(0.25 g) 63	225 – 230	5.21 ± 0.35
$[Cu(H_2L)(H_2O) (NO_3)]$ (2)	Deep green	(0.24 g) 62	195 – 200	18.57 ± 0.35
$[Ni(H_2L) (NO_3)].2 (H_2O)$ (3)	Reddish brown	(0.26 g) 66	115 – 120	15.07 ± 0.83

Table S3: Metal–ligand formation constants and stoichiometric

		Stability constant (K) at Temp. (^oC)							
	M: H_3L ratio	25 ^oC	LogK	30 ^oC	LogK	37 ^oC	LogK	40 ^oC	LogK
1	1:1	2.38×10^6	6.3761	2.37×10^6	6.3742	2.34×10^6	6.3697	2.34×10^6	6.3697
2	1:1	2.97×10^6	6.4723	2.93×10^6	6.4682	2.94×10^6	6.4683	2.94×10^6	6.4683
3	1:1	3.81×10^5	5.5812	3.79×10^5	5.5787	3.80×10^5	5.5803	3.80×10^5	5.5803

Table S4: FTIR data of the ligand and its Zn(II), Cu(II) and Ni(II) complexes. The B3LYP-GD3/6-311++G**/LanL2DZ calculated results are presented in parenthesis.[a]

	ν(O-H)	ν (N-H)	Im ν(C=N)	Ql ν(C=N)	δ(O-H)	$\nu(NO_3)$[c]	ν(C-O)[d]	ν(M-O)	ν(M-N)
**H_3L**	3368 (3564)	3275 (3359)	1639 (1579)	1620 (1532)	1411 (1323)	-	1055 (987)	-	-
**1**	3349 (3575)	3294 (3374)	1657 (1680)	1633 (1608)	1446 (1362)	-	1034 (1054)	524 (490)	460 (464[e], 336[f])
**2**	3664-3334 (3734, 3713[b])	3168 (3374)	1652 (1661)	1627 (1586)	1460 (1426,),	1380 (1381, 1301, 1052)	1059 (1044)	626 (703, 466)	474 (467[e], 386 [f])
**3**	3673-3373 (3736, 3574 [b])	3197 (3088)	1650 (1669)	1634 (1593)	1439 (1421)	1351 (1457, 1295,	1037 (1038)	534 (750, 563)	462 (503[e], 470[f])

[a] stretching and bending vibrational modes of the free ligand and its complexes, unless stated otherwise. Im = Imine, Ql = Quinoline

[b] O-H stretching frequency of a water ligand

[c] the B3LYP calculated results represent for the stretching frequencies of the three N-O bonds.

[d] C-O vibrational frequency involving O-bonded to the metal center.

[e] vibrational frequency involving amine (NH) bonded to the metal.

[f] vibrational frequency involving imine (C=N) bonded to the metal.

Table S5: Estimated lattice parameters (a, b, c, α, β and γ) of the complexes **1** –**3**.

	**1**	**2**	**3**
Empirical formula	$C_{14}H_{16}ClN_3O_2Zn$	$C_{14}H_{18}CuN_4O_6$	$C_{14}H_{20}N_4NiO_7$
Formula weight	359.13	401.86	415.02
Temperature	298 K	298 K	298 K
Wavelength (A°)	1.5406	1.5406	1.5406
Crystal system	Tetragonal	Orthorhombic	Orthorhombic
Unit cell			
a (A°)	7.2843	21.7992	13.6128
b (A°)	7.2437	3.8586	7.8610
c (A^o)	6.8345	8.6055	7.0192
Average crystallite size(A^o)	27.86	33.54	37.40
2θ (^{o})	5–80	5–80	5–80

Table S6: Powder XRD data and estimated Miller indices (hkl) of the complexes **1**–**3**.

Peak No.	2θ	θ	Sinθ	$h^2+k^2+l^2$	d-spacing(A^o)	h	k	l
			Powder XRD data for Zn complex					
1	8.58124	6.47145	0.187154532	1	6.83447	0	0	1
2	13.4596	6.7298	0.431914757	2	6.57324	0	-1	1
3	17.2018	8.6009	0.733785952	2	5.15076	1	1	0
4	17.7436	8.8718	0.525223692	5	4.99468	-2	0	1
5	23.5714	11.7857	0.70375601	4	3.77133	2	0	0
6	24.4995	12.24975	0.311356938	5	3.63052	-1	0	2
7	25.5	12.75	0.182599135	4	3.4903	0	0	2
8	25.9936	12.9968	0.417261061	9	3.42513	-1	-2	2
9	26.6016	13.3008	0.670163747	5	3.34821	-1	2	0
10	27.1074	13.5537	0.83455766	5	3.28687	2	1	0
			Powder XRD data for Copper complex					
1	8.1052	4.0526	0.790121594	4	10.89961	2	0	0
2	12.0577	6.02885	0.251602154	5	7.33415	2	0	1
3	16.0819	8.04095	0.982572273	16	5.50683	4	0	0
4	16.6911	8.34555	0.88159366	17	5.30719	4	0	1
5	20.626	10.313	0.77595145	4	4.30274	0	0	2
6	22.1247	11.06235	0.99777133	8	4.01455	2	0	2
7	22.9172	11.4586	0.894705139	37	3.87748	6	0	1
8	25.6083	12.80415	0.235545075	3	3.47578	1	1	1
9	25.8894	12.9447	0.369368512	6	3.43868	2	1	1
10	26.1886	13.0943	0.503745718	11	3.40007	3	1	1
11	28.8848	14.4424	0.953776941	18	3.08853	4	1	1
12	29.1844	14.5922	0.8982465	40	3.0575	6	0	2
			Powder XRD data for Nickel complex					
1	11.9469	5.97345	0.30480655	1	7.40192	0	0	1
2	12.2563	6.12815	0.154414983	2	7.21575	-1	0	1

3	12.9965	6.49825	0.213410631	4	6.8064	2	0	0
4	15.2427	7.62135	0.973063071	2	5.80807	1	0	1
5	16.023	8.0115	0.987619613	3	5.52694	-1	1	1
6	18.2327	9.11635	0.303561154	6	4.86178	-2	1	1
7	19.9455	9.97275	0.520957269	4	4.44798	0	2	0
8	24.1131	12.05655	0.488020681	6	3.68782	-1	2	1
9	24.6311	12.31555	0.248198979	5	3.61142	0	-1	2
10	28.0363	14.01815	0.99292584	8	3.18005	0	-2	2

Table S7: Radical scavenging activity of the synthesised compounds (mean ± SD)

Conc.	Ligand(H_3L)	1	2	3	Ascorbic acid
115	55.5 ± 0.32	94.19 ± 0.45	93.96 ± 0.25	60.44 ± 0.34	97.71 ± 0.39
100	55.39 ± 0.13	93.65 ± 0.22	93.24 ± 0.41	60.09 ± 0.26	97.23 ± 0.37
85	54.61 ± 0.16	91.68 ± 0.19	82.7 ± 0.17	58.75 ± 0.45	96.25 ± 0.05
70	53.8 ± 0.32	82.9 ± 0.09	74.56 ± 0.25	58.21 ± 0.25	91.8 ± 0.34
55	52.74 ± 0.13	80.67 ± 0.39	63.8 ± 0.13	55.67 ± 0.20	87.85 ± 0.13
40	51.66 ± 0.27	75.63 ± 0.30	55.51 ± 0.36	48.49 ± 0.33	75.55 ± 0.49
25	48.23 ± 0.18	51.72 ± 0.15	54.62 ± 0.30	48.18 ± 0.33	60.89 ± 0.92
10	47.07± 0.23	44.04± 0.81	53.97 ± 0.11	47.85 ± 0.19	47.35 ± 0.23
5	46.99 ± 0.40	43.67± 0.29	53.86 ± 0.20	47.8 ± 0.05	41.67 ± 0.65
IC_{50} (μg/ml)	35.36	10.46	8.62	27.56	4.49

Table S8: ADME and Drug likeness descriptors of Ligand and its complexes.

A. Physicochemical properties

359.13	401.86	415.02

Molecule	MW	HBAs	HBDs	TPSA	iLOGP	Lipinski #violations
H_3L	259.3	4	3	77.74	2.22	0
1	359.13	4	2	66.74	0	0
2	401.86	8	3	142.02	0	0
3	415.02	7	2	121.79	0	0
Cipro	331.34	5	2	74.57	2.24	0

B. Pharmacokinetic properties

Molecule	GI absorption	BBB permeant	Pgp substrate	CYP1A2 inhibitor	CYP2C19 inhibitor	CYP2C9 inhibitor	CYP2D6 inhibitor	CYP3A4 inhibitor	log Kp (cm/s)
H_3L	High	No	Yes	No	No	No	No	No	-7.38
1	High	Yes	Yes	Yes	No	No	Yes	Yes	-7.1
2	Low	No	Yes	No	No	No	No	No	-7.53
3	High	No	Yes	No	No	No	No	No	-7.45
Cipro	High	No	Yes	No	No	No	No	No	-9.09

Table S9: Molecular docking of ligand and complexes against *E. Coli* DNA Gyrase (PDB ID 6f86).

S. No.	Binding Affinity (kcal/mol)	H-bond	Residual interactions	
			Hydrophobic/Pi-Cation	Van der Waals
H_3L	-7.1	Asp-73, Gly-77, Thr-165	Val-43, Ile-94, Ile-78	Ala-47, Asn-46, Glu-50, Pro-79
1	-6.5	Asp-73, Thr-165	Ala-47, Val-43	Gly-77, Arg-76, Glu-50, Asn-46, Ile-78, Gly-164
2	-6.8	Asp-73, Asn-46, Gly-77	Arg-76, Pro-79,	Glu-50
3	-6.3	Thr-165	Ile-78	Asp-73, Ala-47, Asn-46, Glu-50, Gly-77, Pro-79

Table S10: Molecular docking scores and residual amino acid interactions of metal complexes against *P. aeruginosa* LasR.DNA binding domain (PDB: 2UV0).

S. No.	Cpds.	Affinity (kcal/mol)	H-bond	H-Bond distance (Å)	Residual Hydrophobic/Pi-Cation/Pi-Anion/ Pi-Alkyl interactions
1	1	– 7.3	Ser-129	2.50	Asp-73, Tyr-64, Leu-36, Trp-88, Phe-101, Leu-110, Ala-70, Gly-38, Cys-79, Ile-52, Thr-115, Ala-127, Thr-75, Tyr-56, Tyr-93
2	2	– 8.2	Tyr-47	2.04	Asp-73, Tyr-64, Leu-36, Arg-61, Thr-75, Ser-129, Tyr-56, Trp-60, Ile-52, Ala-50, Gly-38, Ala-70, Leu-39
3	3	– 8.2	Tyr-47	3.05	Asp-73, Tyr-64, Leu-36, Tyr-56, Ala-127, Gly-38, Ala-50, Ile-52, Asp-65, Arg-61, Leu-39, Trp-60, Trp-88, Thr-75, Ser-129
4	H_3L	– 7.8	Tyr-64	2.55	Asp-73, Trp-88, Phe-101, Ala-105, Leu-110, Trp-60, Tyr-93, Tyr-56, Thr-75, Ser-129, Thr-115, Arg-61
5	Cipro.	– 8.0	Asp-73, Trp-60, Tyr-47,	2.45, 2.50, 2.55	Tyr-64, Leu-36, Val-76, Ala-127, Cys-79, Ala-50, Gly-38, Thr-115, Trp-88, Thr-75, Tyr-56, Leu-110, Phe-101, Arg-61, Ala-70

$$K' = \frac{\left[{}^{A_2}/_{A_1}\right]}{\left[1 - {}^{A_2}/_{A_1}\right]\left[1 - {}^{A_2}/_{A_1}\right]} \quad (S1)$$

Where, A_1 = absorbance at extrapolated point, A_2 = actual absorbance

Table S11: B3LYP-GD3/311++G(d,p)/LanL2DZ/PCM/methanol optimized geometries

Complex 1; *G* = -1383.589916 hartree

C	-5.069064000	-0.815903000	0.908090000
C	-3.700692000	-0.982695000	0.827247000
C	-2.821682000	0.112080000	1.013307000
C	-3.417334000	1.384422000	1.276206000
C	-5.565473000	0.534045000	1.090735000
H	-0.966318000	-0.978077000	0.762430000
H	-3.287765000	-1.972646000	0.649336000
C	-1.407756000	-0.006795000	0.968493000
C	-2.577948000	2.502649000	1.505503000
C	-1.203187000	2.358841000	1.462248000
C	-0.611446000	1.099815000	1.188501000
H	-3.039795000	3.464316000	1.706041000
H	-0.566030000	3.221011000	1.635893000
H	0.469800000	1.009885000	1.154232000
N	-4.774357000	1.562454000	1.279627000
N	-6.962087000	0.782557000	1.032240000
C	-5.929448000	-2.001949000	0.827451000
H	-5.518349000	-2.873952000	0.305355000
N	-7.089455000	-2.041572000	1.364987000
C	-7.977601000	-3.192227000	1.263992000
H	-7.893120000	-3.758377000	2.200665000
C	-9.418643000	-2.637046000	1.157914000
C	-7.390227000	2.187956000	0.815278000
H	-8.394482000	2.140788000	0.385945000
C	-7.450013000	3.036848000	2.084578000
O	-8.590686000	2.737187000	2.892727000
H	-8.311198000	2.127526000	3.597577000
H	-6.721808000	2.670880000	0.093273000
H	-6.525306000	2.927486000	2.658295000
H	-7.537236000	4.085093000	1.776637000
O	-9.697642000	-1.717453000	2.178465000
H	-9.527127000	-2.180853000	0.154986000
H	-10.111313000	-3.494132000	1.198697000
H	-7.716753000	-3.846876000	0.422010000
Zn	-8.148981000	-0.619548000	2.658885000
Cl	-7.400081000	0.370753000	4.656358000
H	-7.374944000	0.189982000	0.314884000

Complex 2; *G* = -1410.618846 hartree

C	-5.508571000	-0.690127000	-0.525842000
C	-4.148056000	-0.653416000	-0.781533000
C	-3.379983000	0.489998000	-0.461552000
C	-4.067618000	1.585311000	0.148837000
C	-6.113730000	0.518148000	-0.013714000
H	-1.464937000	-0.265772000	-1.138577000
H	-3.651452000	-1.530969000	-1.187643000
C	-1.978267000	0.571269000	-0.673820000
C	-3.332046000	2.728554000	0.544708000
C	-1.966849000	2.780012000	0.330104000
C	-1.284519000	1.699499000	-0.284583000
H	-3.864048000	3.553447000	1.007753000
H	-1.407341000	3.659992000	0.632933000
H	-0.212888000	1.762940000	-0.444845000
N	-5.424340000	1.573798000	0.329150000
N	-7.538510000	0.575428000	0.125068000
C	-6.181705000	-1.981643000	-0.643737000
H	-5.620353000	-2.764804000	-1.163080000
N	-7.305993000	-2.269075000	-0.097360000
C	-7.819638000	-3.654029000	-0.077310000
H	-7.140590000	-4.251111000	0.542330000
C	-9.207679000	-3.601441000	0.575462000
C	-8.130738000	1.873508000	0.532487000
H	-7.607151000	2.225678000	1.420776000
C	-8.096391000	2.928112000	-0.572122000
O	-8.802312000	4.063101000	-0.058165000
H	-8.774854000	4.770528000	-0.715842000
H	-9.170044000	1.678343000	0.794963000
H	-7.063628000	3.188198000	-0.826619000
H	-8.594457000	2.534542000	-1.470636000
O	-10.186444000	-0.469696000	-0.373809000
O	-9.166247000	-2.787018000	1.724158000
H	-9.935373000	-3.216327000	-0.159331000
H	-9.521579000	-4.621017000	0.847466000
H	-7.840935000	-4.075746000	-1.089106000
O	-9.385981000	-0.106509000	2.624602000
H	-10.223193000	0.176776000	2.153488000
N	-11.292943000	0.068673000	-0.066740000
O	-12.216574000	0.132067000	-0.895148000
O	-11.448492000	0.554787000	1.102141000
Cu	-8.415981000	-1.123651000	1.131866000
H	-9.656887000	-0.781120000	3.266348000
H	-7.972724000	0.274787000	-0.751185000

Complex 3; G = -1460.222670 hartree

C	-5.605098000	-0.784366000	0.301695000
C	-4.271238000	-0.776997000	-0.067679000
C	-3.597632000	0.441759000	-0.311621000
C	-4.351174000	1.646041000	-0.148567000
C	-6.247945000	0.489345000	0.499371000
H	-1.676370000	-0.405865000	-0.848046000
H	-3.742462000	-1.716452000	-0.205637000
C	-2.241470000	0.513903000	-0.726997000
C	-3.732601000	2.891466000	-0.413764000
C	-2.411708000	2.933088000	-0.820529000
C	-1.660130000	1.740636000	-0.977101000
H	-4.315128000	3.798095000	-0.285277000
H	-1.940278000	3.890194000	-1.022394000
H	-0.624224000	1.797749000	-1.296047000
N	-5.652184000	1.630632000	0.278858000
N	-7.593346000	0.520116000	1.015484000
C	-6.293256000	-2.061652000	0.398207000
H	-5.669744000	-2.958079000	0.412931000
N	-7.570743000	-2.193978000	0.410507000
C	-8.211498000	-3.518814000	0.417953000
H	-8.634253000	-3.672142000	1.416609000
C	-9.347389000	-3.386225000	-0.597866000
C	-8.158090000	1.880476000	1.257921000
H	-7.507675000	2.423820000	1.949753000
C	-8.390667000	2.691812000	-0.016307000
O	-9.491041000	3.573120000	0.254631000
H	-9.543547000	4.224876000	-0.456710000
H	-9.124398000	1.712390000	1.734766000
H	-7.497249000	3.260745000	-0.282926000
H	-8.630348000	2.014833000	-0.844173000
O	-10.377137000	0.384039000	0.087844000
O	-10.035605000	-2.187750000	-0.326666000
H	-8.918819000	-3.390946000	-1.614897000
H	-10.032590000	-4.242451000	-0.513396000
H	-7.491753000	-4.314650000	0.194864000
O	-7.533075000	-0.873370000	3.512591000
H	-7.558628000	-0.332944000	4.314777000
O	-7.467684000	-0.178342000	-2.773322000
H	-8.365022000	0.003852000	-2.439034000
N	-10.877120000	0.685482000	-1.069132000
O	-10.197404000	0.512713000	-2.105371000
O	-12.017385000	1.165784000	-1.096498000

Ni	-8.882238000	-0.849533000	0.207617000
H	-6.916067000	0.527619000	-2.413305000
H	-8.305960000	-1.453365000	3.567637000
H	-7.562816000	0.046599000	1.940219000

Ligand, *G* = -858.271370 hartree

C	-5.182665000	-0.672763000	1.267565000
C	-3.826400000	-0.916332000	1.188174000
C	-2.894375000	0.141865000	1.053851000
C	-3.426695000	1.470920000	1.005767000
C	-5.617478000	0.719760000	1.215099000
H	-1.107356000	-1.079738000	1.007968000
H	-3.462552000	-1.940959000	1.227814000
C	-1.492159000	-0.063675000	0.968590000
C	-2.519516000	2.556247000	0.869582000
C	-1.158284000	2.327831000	0.787835000
C	-0.634033000	1.010742000	0.837748000
H	-2.923578000	3.563530000	0.831932000
H	-0.478151000	3.168773000	0.684339000
H	0.437998000	0.852447000	0.772638000
N	-4.762169000	1.730510000	1.089839000
N	-6.945638000	0.997944000	1.298735000
C	-6.086450000	-1.815250000	1.404684000
H	-5.584391000	-2.792653000	1.457564000
N	-7.365993000	-1.733253000	1.466736000
C	-8.138065000	-2.961408000	1.594898000
H	-8.662544000	-2.935229000	2.558460000
C	-9.182281000	-3.044712000	0.478293000
C	-7.500865000	2.343224000	1.273310000
H	-8.561007000	2.250383000	1.020334000
C	-7.359073000	3.058058000	2.620674000
O	-8.018104000	2.348370000	3.677284000
H	-7.606277000	1.474559000	3.746062000
H	-7.008299000	2.937402000	0.495997000
H	-6.296660000	3.198249000	2.854615000
H	-7.837897000	4.040258000	2.565523000
O	-10.125442000	-1.970756000	0.533810000
H	-8.677599000	-3.065589000	-0.498365000
H	-9.759816000	-3.967150000	0.586645000
H	-7.504345000	-3.862304000	1.568742000
H	-7.558021000	0.188494000	1.382198000
H	-9.628022000	-1.141540000	0.489939000
